# Distinct serum and cerebrospinal fluid metabolic signatures associate with pain and fatigue in knee osteoarthritis

**DOI:** 10.1016/j.bbih.2026.101227

**Published:** 2026-03-27

**Authors:** Aline U. Bjerkhaug, Jenny E. Jakobsson, Aisha S. Ahmed, Henrik Carlsson, Ida Erngren, Asma Al-Grety, Alex Bersellini Farinotti, Camilla I. Svensson, Kim Kultima, Eva Kosek

**Affiliations:** a Department of Clinical Neuroscience, Karolinska Institutet, Stockholm, Sweden; b Department of Surgical Sciences, Uppsala University, Uppsala, Sweden; c Department of Medical Sciences, Uppsala University, Uppsala, Sweden; d Department of Molecular Medicine and Surgery, Center for Molecular Medicine, Karolinska Institutet, Stockholm, Sweden; e Department of Physiology and Pharmacology, Center for Molecular Medicine, Karolinska Institutet, Stockholm, Sweden

**Keywords:** Osteoarthritis, Metabolomics, Serum, Cerebrospinal fluid, Pain mechanisms, Branched-chain amino acids

## Abstract

Pain is the primary reason patients with knee osteoarthritis (OA) seek surgical intervention when pharmacological treatments lose efficacy. The pain arises from complex mechanisms involving peripheral and central sensitization and inflammatory processes. Emerging research links altered metabolites and lipids, such as lysophosphatidylcholine 16:0 and polyunsaturated fatty acids, to OA pathophysiology and pain modulation. Metabolomic studies in serum and synovial fluid have revealed potential biomarkers and novel therapeutic targets related to pain severity. This study aims to identify metabolic signatures in serum and cerebrospinal fluid (CSF) from knee OA patients, explore systemic and central metabolic interactions, and examine associations with symptom severity.

The study included 36 knee OA patients, 38 healthy controls (HC) for serum analysis, and a separate non-healthy CSF control group of 39 individuals with non-inflammatory neurological symptoms (NINS). Samples were subjected to two targeted metabolomics methods using liquid chromatography high resolution mass spectrometry. Participants completed detailed questionnaires assessing pain intensity, fatigue, sleep disturbances, depression, anxiety, and functional disability.

HC exhibited higher levels of 11 amino acids (including branched-chain amino acids, histidine, and tryptophan), five bile acids, and four lipids in serum. Conversely, OA patients showed elevated uracil concentrations. Notably, bile acids, glycocholic acid, and glycochenodeoxycholic acid were positively correlated with pain and symptom severity, despite being reduced in OA patients. Lower levels of alanine, isoleucine, and short-chain acylcarnitines (CARs 3:0, 4:0, and 5:0) were associated with higher fatigue ratings. Nucleotides and their derivatives correlated with knee pain intensity, pressure pain sensitivity, and fatigue.

In CSF, OA patients generally had increased metabolite levels compared to NINS controls, such as 3-hydroxyphenylacetic acid, histamine, indole, and glutarylcarnitine. Histamine and 3-hydroxyphenylacetic acid were also significantly associated with pain intensity, sleep disturbance, and pressure pain sensitivity. Furthermore, numerous CSF metabolites showed moderate to high correlations with serum levels.

This study reveals serum and CSF metabolic changes in knee OA patients linked to symptoms like pain and fatigue. It highlights complex systemic and central metabolism interactions and identifies potential biomarkers for personalized OA treatment, advancing clinical understanding.

## Graphical abstract

**Figure:**
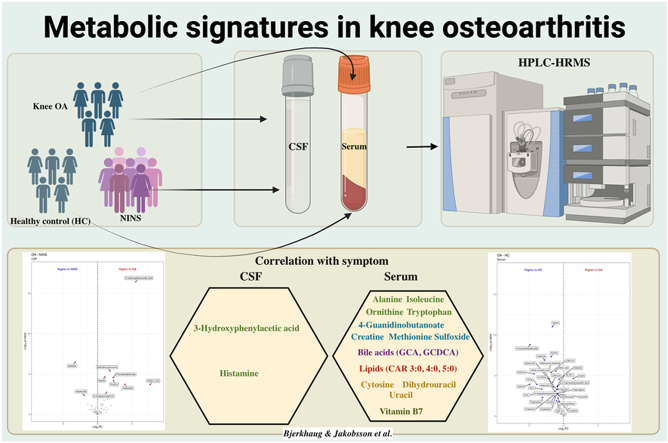

## 1 Introduction

Pain is the most prominent symptom and remains the primary reason patients with osteoarthritis (OA) seek total knee replacement (TKR) (Eitner et al., 2017; Fu et al., 2018; Sharma, 2021). The mechanisms underlying OA pain are complex, involving peripheral (Schaible and Grubb, 1993; Hawker et al., 2008) and central sensitization (Kosek and Ordeberg, 2000a, 2000b; Arendt-Nielsen et al., 2010) and inflammatory processes (Miller et al., 2014; Sandström et al., 2024). The neuroimmune interface plays a crucial role in chronic pain (Grace et al., 2014), wherein pro-inflammatory cytokines have been demonstrated to contribute to nociception in both preclinical models and clinical cohorts of OA (Miller et al., 2014; Hill et al., 2007; Dainese et al., 2022). Pro-inflammatory cytokines, including tumour necrosis factor (TNF), interleukin (IL)-6, and IL-8, can sensitize peripheral nociceptive afferents (Brenn et al., 2007; Richter et al., 2010) and are associated with pain severity in knee OA (Kosek et al., 2018). However, cytokines may serve different functions depending on the biological compartment. This is illustrated by divergent associations between IL-6 and IL-8 and symptom severity in OA patients, positive if the cytokines were assessed in synovial fluid and negative if analysed in cerebrospinal fluid (CSF) (Kosek et al., 2018). Thus, while neuroimmune signalling is traditionally linked to nociception, recent research suggests it may also have analgesic and neuroprotective effects within the central nervous system (CNS) (Palada et al., 2020). These findings highlight the dual role of neuroinflammation in OA, suggesting it may contribute to pain and symptom relief. Furthermore, emerging evidence indicates that both peripheral and spinal mechanisms maintain chronic OA pain. Gowler et al. (2020) showed that brain-derived neurotrophic factor (BDNF) and its receptor, tropomyosin receptor kinase B (TrkB), are expressed in OA synovium, with peripheral BDNF/TrkB signaling increasing OA pain in rodent models (Gowler et al., 2020). Additionally, a rat OA model revealed activation of glial cells in the dorsal root ganglia (DRG) and spinal cord during chronic OA, marked by increased glial fibrillary acidic protein (GFAP) and ionized calcium-binding adapter molecule 1 (IBA-1) expression (Adães et al., 2017). Blocking glial activation reduced pain, suggesting spinal neuroimmune involvement (Adães et al., 2017). Together, these findings imply that OA pain originates peripherally but is sustained by neuroimmune processes in the DRG and spinal cord, resembling central sensitization.

Despite limited evidence supporting their long-term efficacy, pharmacotherapy using drugs with anti-inflammatory effects, such as opioids (Chou et al., 2015; El-Tallawy et al., 2021) and non-steroidal anti-inflammatory drugs (NSAIDs) (Temple et al., 2006; Sawitzke et al., 2010), remains commonly used for knee OA. However, they are associated with significant risks, including addiction, hepatotoxicity, and cardiovascular complications. Injectable therapies and surgical interventions may offer greater pain relief in advanced cases. Still, their use is also limited by potential adverse effects, making surgery the most effective option for severe osteoarthritis despite ongoing exploration of alternative treatments (Sharma, 2021; Charlesworth et al., 2019). Advancing biomarkers and understanding pain pathways are crucial for optimizing OA treatment (Zhai et al., 2018). Analysing individual lipid profiles and identifying specific biomarkers could support early diagnosis of OA. Lysophosphatidylcholine (LPC) 16:0 in synovial fluid has been implicated in chronic joint pain by activating acid-sensing ion channel 3 (ASIC3) (Jacquot et al., 2022). Synovial LPC 16:0 has also shown a positive correlation with circulating lipid and lipoprotein markers, including cholesterol, high-density lipoprotein (HDL), and low-density lipoprotein (LDL), in patients with knee OA (Khoury et al., 2023). A meta-analysis of two studies identified several metabolites in serum, including acylornithine, carnosine, cortisol, cortisone, cystine, DOPA, glycolithocholic acid sulphate (GLCAS), phenylethylamine, and succinic acid as significantly associated with pain scores. Additionally, inflammatory cytokines, including IL-10, IL-13, IL-1β, IL-2, IL-8, and TNF, were linked with these pain-associated metabolites, although the potential causal links remain to be established (Mehta et al., 2023). Jónasdóttir et al. detected seven polyunsaturated fatty acids (PUFAs) in the synovial fluid of OA patients (Jónasdóttir et al., 2017). Studies have demonstrated alterations in human plasma and serum lipid metabolism that could distinguish OA patients from healthy individuals and stratify disease severity, highlighting disruptions in fatty acid and lipid-related metabolic pathways (Castro-Perez et al., 2010) (Zhang et al., 2015).

Several studies (Gruenwald et al., 2009; Hesslink et al., 2002; Kraemer et al., 2004, 2005) have explored the impact of fatty acid supplementation on knee OA, with cetylated fatty acids (CFA) showing benefits such as improved knee flexion and modest gains in patient-reported outcomes. The topical CFA application also enhanced the range of motion over 30 days. However, unclear placebo formulations limit the reliability of these findings. In parallel, recent research by Yang et al. (2025) revealed distinct bile acid profiles in plasma associated with OA and gut microbiota. It demonstrated the effectiveness of a bile acid receptor-targeting drug in reducing OA symptoms in mice. Human data from the same study by Yang et al. further suggest this drug may lower the risk of knee replacement, supporting its potential for clinical repurposing (Yang et al., 2025). These results indicate that metabolic changes in OA may be involved in pathological mechanisms and hence could become a new target in developing new treatment strategies.

This cross-sectional study aimed to identify metabolic signatures in serum and CSF among individuals with knee OA compared to controls, asses their relationship with OA symptoms severity, and explore interactions between systemic and central metabolite concentrations.

## 2 Materials and methods

### 2.1 Study design and overview

This study utilizes a cohort that has been previously investigated, with published circulating cytokine levels and tissue-specific gene expression (Kosek et al., 2018; Palada et al., 2020, 2021; Jacquot et al., 2022; Ahmed et al., 2018). Demographic characteristics of the patient cohort are detailed in Table 1. A total of 36 OA individuals were analysed. Out of these, 36 had CSF and 32 had serum collected. Additionally, 38 healthy controls (HC) were included, phenotyped, and had serum samples taken. For ethical reasons, we had to rely on 39 non-healthy CSF controls with non-inflammatory neurological symptoms (NINS), see below.

**Table 1:** Participant characteristics for individuals with knee osteoarthritis (OA) and control subjects. The samples are presented as a total number of participants and a number of serum and cerebrospinal fluid (CSF) samples. The sex is presented as the number of females (F) and males (M). Samples from 36 individuals with OA were included, of whom 32 provided serum samples and 36 provided cerebrospinal fluid (CSF). Visual analogue scale (VAS) scores for knee pain were 0 in healthy controls and therefore not reported.

Factor	OA (n = 36) Mean (Range)	Healthy Controls (n = 38) Mean (Range)	NINS (n = 39) Mean (Range)	OA vs. HC *p*-value	OA vs. NINS *p*-value
Sex (F/M)	15/21	19/19	23/16	0.6272	0.2053
Age (years)	64.7 (49–73)	64.8 (49–73)	47.2 (26–73)	0.7904	8.018 × 10^−8^
BMI (kg/m^2^)	27.8 (21.6–36.3)	24.9 (19.6–31.6)	—	0.00018	—
VAS – Average Pain	48.8 (7–100)	2.1 (0–13)	—	2.15 × 10^−13^	—
VAS – Current Pain	40.4 (0–100)	2.2 (0–24)	—	1.37 × 10^−10^	—
VAS – Knee Pain	16.3 (0–75)	—	—	—	—
PSQI (Sleep Quality)	8.4 (3–15)	3.7 (0–12)	—	1.40 × 10^−6^	—
KOOS (Knee-related disability)	37.2 (7.7–57.8)	98.7 (81.2–100)	—	1.39 × 10^−14^	—
MFI – Total	50.1 (25–73)	33.4 (24–60)	—	3.34 × 10^−8^	—
MFI – General Fatigue	11.5 (5–16)	6.0 (4–11)	—	2.58 × 10^−10^	—
HAD – Anxiety	3.7 (0–9)	1.6 (0–8)	—	0.00057	—
HAD – Depression	3.4 (0–8)	1.2 (0–7)	—	1.16 × 10^−5^	—
PPT – Average (kPa)	487.7 (150.2–863.5)	460.8 (242.8–915)	—	0.4509	—
PPT – Knee (kPa)	344.5 (131–589)	448.4 (197–817)	—	0.005715	—

Abbreviations: BMI, body mass index; HAD, Hospital Anxiety and Depression Scale; KOOS, Knee Injury and Osteoarthritis Outcome Score; MFI TOT, total score from Multidimensional Fatigue Inventory; MFI GF, general fatigue subscale from Multidimensional Fatigue Inventory; PPT, Pressure Pain Threshold; PSQI, Pittsburgh Sleep Quality Index.

Typically, within a week before surgery, patients completed questionnaires and assessed their pressure pain sensitivity by pressure algometry. On the day of surgery, before the procedure, venous blood samples were drawn from the antecubital vein, and CSF was collected via lumbar puncture before administering spinal anaesthesia. Healthy controls were assessed following the same protocol as OA patients with questionnaires, pressure algometry, and intravenous blood samples collected from the antecubital vein.

The study was approved by the regional ethical review board in Stockholm, Sweden (reference number 2011/2036-31-1) and was conducted in compliance with the principles outlined in the Declaration of Helsinki. All participants were thoroughly informed about the study procedures, and written informed consent was obtained.

### 2.2 Subjects

#### 2.2.1 OA patients

The inclusion criteria for patient enrollment were individuals aged between 25 and 75 years, radiologically confirmed knee OA, and knee pain as the predominant symptom and primary indication for surgical intervention. Exclusion criteria encompassed chronic pain conditions unrelated to knee OA, including fibromyalgia, degenerative disc disease, disc herniation, inflammatory rheumatic diseases, neurological diseases, and a history of prior knee surgery. They were recruited consecutively from the waiting list for total knee replacement at Ortho Center, Upplands Väsby, Sweden.

Data on medication use were collected for all patients. The reported medications included acetaminophen, analgesics (codeine, tramadol, buprenorphine patches, and ketobemidone), and nonsteroidal anti-inflammatory drugs (NSAIDs), the latter being discontinued 14 days before surgery. As part of preoperative management, all patients received 2 g of acetaminophen (paracetamol) and 10 mg of oxycodone orally.

#### 2.2.2 Healthy controls

Age- and sex-matched HC were recruited via advertisement in local newspapers. They were prescreened by telephone and once more during the examination day regarding exclusion criteria, which were the same as those for OA patients. Additionally, they were also ensured not to suffer from OA or weakly average pain intensity >20 mm on a 100 mm visual analogue scale (VAS).

#### 2.2.3 Cerebrospinal fluid controls

For ethical reasons, a separate control group was used for CSF. Cerebrospinal fluid samples were collected from 39 individuals with non-inflammatory neurological symptoms (NINS) who were evaluated for neurological symptoms, mostly paresthesias, at the Department of Neurology, Karolinska University Hospital. Clinical assessments, including blood tests, CSF analysis, and brain MRI, showed no signs of inflammatory conditions. None of the participants were on regular medications or took analgesics on the day of sample collection. Aside from headaches, no other pain disorders were reported, and all participants consented to the use of their CSF samples for research.

### 2.3 Questionnaires

Pain intensity on the examination day was assessed using a 100 mm visual analogue scale (VAS) for average (VAS average), current (VAS now), and knee pain (VAS knee), where 0 indicated “no pain” and 100 represented “worst imaginable pain”. Sleep was assessed using the Pittsburgh Sleep Quality Index (PSQI) (Buysse et al., 1989), a 19-item measure of sleep quality over the past month (0–21, higher scores indicating poorer sleep). Overall symptom severity was assessed using the Knee Injury and Osteoarthritis Outcome Score (KOOS) (Roos et al., 1998, Roos and Toksvig-Larsen, 2003), comprising five subscales: pain, symptoms, daily activities, sport/recreation function, and knee-related quality of life. Each subscale was scored from 0 (worst) to 100 (best), with an overall average calculated for analysis. Fatigue was evaluated using the Multidimensional Fatigue Inventory (MFI-20) (Hagelin et al., 2007; Ericsson and Mannerkorpi, 2007; Lin et al., 2009), a 20-item tool assessing five dimensions of fatigue, general fatigue (GF), physical fatigue (PF), mental fatigue (MF), reduced motivation (RM), and reduced activity (RA) with subscores ranging from 4 to 20 (higher scores indicating greater fatigue). Here, we analysed MFI-20 total score (MFI_TOT; sum of all five dimensions) and MFI-20 general fatigue (MFI_GF). Psychological assessments included the Hospital Depression and Anxiety Scale (HADS) (Bjelland et al., 2002), a psychometric tool for non-psychiatric patients with anxiety and depression subscales (0–21, higher scores indicating greater severity).

### 2.4 Pressure algometry

A specially trained, experienced, investigator (Aisha Ahmed) performed all pressure algometry assessments. The pressure algometer (Somedic Sales AB, Hörby, Sweden) used in this study had a circular metallic probe area of 1 cm^2^, covered by a 2 mm thick neoprene rubber to avoid adverse skin stimuli due to sharp metal edges. The rate of pressure increase was kept to approximately 40–60 kPa, with the help of continuous visual feedback (Kosek et al., 1993).

The participants were instructed to indicate pressure pain thresholds (PPTs) by pressing a push-button as soon as the pressure sensation became painful, which froze the current pressure value on the display. Before the examination of each subject, the pressure algometer was checked for accuracy using a 1 kg weight (accepting values between of 96-104 kPa). PPTs were assessed at the medial epicondyle of the femur, near the knee joint space (PPT_knee). PPTs were also recorded bilaterally at the trapezius muscle (midpoint of the upper border) and the gluteal muscle (upper outer quadrant of the buttocks at the anterior muscle fold) to assess overall pain sensitivity. The average of these assessments (PPT_average) was calculated for each participant.

### 2.5 Sample collection and storage

Intravenous blood was drawn into two 8.5 mL BD Vacutainer® SST II plastic tubes, incubated at room temperature for 30–40 min, and centrifuged at 2500 rpm for 10 min. The resulting serum was collected, aliquoted, and stored at −80 °C for later analysis.

CSF was collected without additives, immediately centrifuged at 2500 rpm at room temperature, and the supernatant was aliquoted and frozen at −80 °C for future analysis.

### 2.6 Albumin quotient

The albumin concentrations in matched serum and CSF samples from OA patients were quantified using enzyme-linked immunosorbent assay (ELISA; Invitrogen, Waltham, MA, USA), following the manufacturer's guidelines. The albumin quotient (AlbQ) was calculated as the ratio between albumin concentrations in CSF and serum, as a measure of the blood-brain barrier (BBB) integrity.

### 2.7 Mass spectrometry

Metabolomic data were collected for CSF and serum using two different high-performance liquid chromatography-high-resolution mass spectrometry (HPLC-HRMS) methods. The first HPLC-HRMS targeted non-polar metabolites, using reverse phase liquid chromatography (RPLC), and has been previously described (Herman et al., 2018). The second method targeted polar metabolites using hydrophilic interaction liquid chromatography (HILIC). All targeted metabolites are listed in Supplementary Table S1.

#### 2.7.1 Sample preparation

Serum samples were thawed on ice and 25 μL was added to 75 μL ice-cold precipitation solution, a methanol (MeOH) solution with added isotopically labeled internal standards (IS). The samples were vortexed for 15 s and left at −20 °C for 60 min, followed by centrifugation for 15 min at 21,100 RCF and 4 °C. The supernatants were transferred to high-performance liquid chromatography (HPLC) vials and stored at −80 °C until analysis.

For the CSF samples, after thawing on ice, 100 μL was added to 300 μL of the same ice-cold precipitation solution as used for serum but diluted tenfold with MeOH. The samples were treated the same way as described for serum samples above, followed by a concentration of samples after the centrifugation. Next, 300 μL of the supernatants were transferred to polypropylene tubes and dried under a gentle stream of nitrogen. The dried samples were then reconstituted in 40 μL of 5% MeOH in H_2_O, transferred to HPLC vials, and stored at −80 °C until analysis.

#### 2.7.2 Mass spectrometry analysis and data processing

The same methods were applied to serum and CSF samples, with differing injection volumes: 2 μL for serum and 5 μL for CSF. A global pooled quality control (QC) sample (QCG) was prepared by collecting 10 μL from each sample. Quality control and blank samples were used to assess the MS experiments. Coefficients of variation (CVs) for all detected metabolites fasting in Supplementary Table S1.

The RPLC method used a C18 column (Accucore aQ RP C18 column, 100 × 2.1 mm, 2.6 μm, Thermo Scientific), while the HILIC method used an Accucore 150 Amide column (100 × 2.1 mm, 2.6 μm, Thermo Scientific). The RPLC employed a 22 min gradient using two mobile phases: H_2_0 with 0.1% formic acid (mobile phase A) and 90% acetonitrile, 10% 2-propanol, with 0.1% formic acid (mobile phase B). The HILIC method utilized an 18.5-min-long gradient, with mobile phase A consisting of 60% acetonitrile, 40% H_2_O, 10 mM ammonium formate, pH 3, and mobile phase B composed of 95% acetonitrile, 5% H_2_O, 10 mM ammonium formate, pH 3. The gradient was initiated by 0% A for 2.5 min, followed by 0 - 100% A for 8.75 min, 100% A for 2 min, returning to 0% A over 0.25 min, followed by re-equilibration at 0% A for 5 min.

Mass spectrometry was performed in positive ionization mode with a resolution of 70,000. Identical MS settings were applied to both methods: the spray voltage was set to 3.5 kV, the capillary temperature was 320 °C, the sheath gas flow rate was 55, the auxiliary gas flow rate was 15, the sweep gas flow rate was 3, the S-lens RF level was 50, and the auxiliary gas heater temperature was 450 °C. The RPLC method collected data within the m/z range 10-900, while the HILIC method collected data within the m/z range 55-820. The HRMS was calibrated weekly, prior to running samples, to sub-ppm mass accuracy. Prior to calibration the ion source and inlet were cleaned and the instrument performance evaluated to achieve optimal response.

Raw MS data were processed using Tracefinder (v.4.1, Thermo Scientific). Metabolites were required to be present in at least 80% of the samples. Chromatographic peaks were evaluated as extracted ion chromatograms using 10 ppm mass filtering, which virtually removed all noise and resulted in easily interpretable peaks. To reduce batch effects and compensate for weekly intensity decline, relative concentrations were normalized by dividing each metabolite's concentration by the median QCG concentration within its respective batch. Batch effects were visualized with principal component analysis for the MS runs respectively (Supplementary Fig. S1).

### 2.8 Statistical analysis

All statistical analysis was performed in R (v.4.3.3). Differences in sex distribution were tested with a chi-square test. The remaining parameters are presented as mean (min – max) and were compared pairwise with a Mann-Whitney *U* test. Linear regression (LR) models were fitted to log_2_-transformed relative serum and CSF metabolite concentrations. For serum metabolites, LR models were adjusted for age, sex, and BMI. For CSF metabolites, the models were adjusted for age and sex, since BMI was not noted for non-healthy CSF control subjects. P-values were adjusted using the Benjamini-Hochberg false discovery rate (FDR) method to account for multiple testing. An FDR-adjusted p-value (q-value) less than 0.05 was considered statistically significant.

The significantly altered metabolites were further investigated for symptom associations using Spearman's rank correlation analysis. Spearman's rank correlation was also used to assess the associations between matched CSF and serum metabolite relative concentrations. Additionally, ratios of CSF to serum metabolite concentrations were correlated with the CSF to serum albumin quotient (AlbQ) to evaluate the association between metabolite concentrations and the integrity of the BBB. A correlation p-value less than 0.05 was considered significant.

Pathway enrichment analysis was performed for the significantly altered metabolites in serum and CSF, respectively, using Metaboanalyst (v.6.0) with Kyoto Encyclopedia of Genes and Genomes (KEGG) pathways and default settings. Pathways with p-values less than 0.05 were considered significantly enriched.

## 3 Results

### 3.1 Demographic data

Individuals with knee OA exhibited significantly higher body mass index (BMI) and reported greater pain intensity than HC (Table 1). They also experienced more pronounced disturbances in sleep, increased limitations in daily functioning, and a marked decline in knee-related disability. These individuals further reported elevated levels of fatigue, anxiety, and depressive symptoms. Although knee OA patients presented with a significantly lower PPT_knee than HC, the PPT_average was not significantly altered between the groups.

### 3.2 Serum metabolite differences between OA patients and HC

**Fig. 1:**
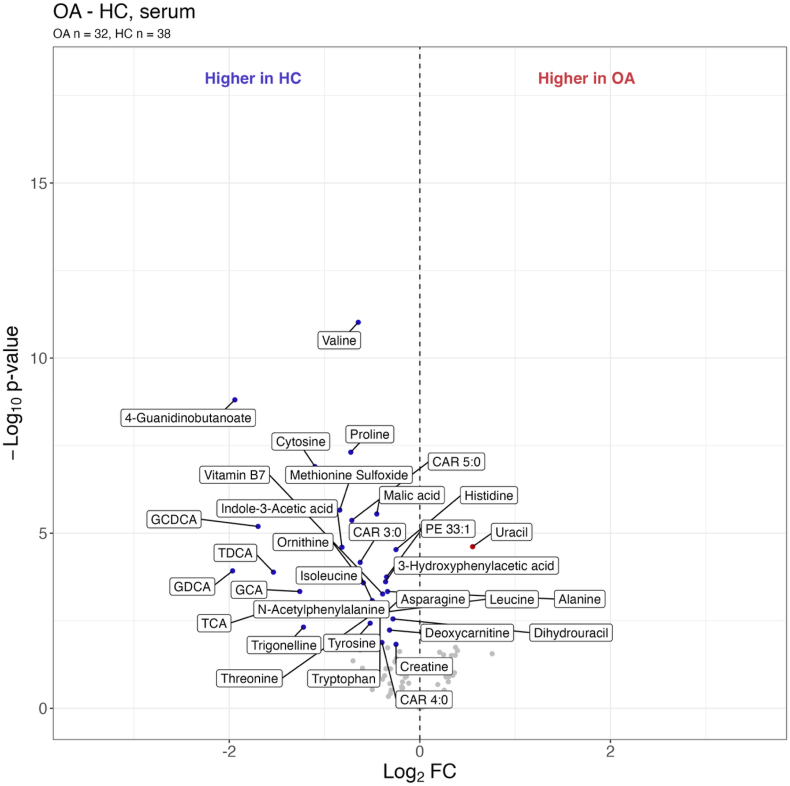
Volcano plot of serum metabolites differing between osteoarthritis (OA) subjects and healthy controls (HC). The x-axis is the log_2_ fold change (FC), and the y-axis shows the -log_10_ of the non-adjusted p-value. The coloured points indicate metabolites with q-values below 0.05. Red points represent metabolites elevated in OA patients compared to HC (positive log_2_ FC), while blue points indicate higher concentrations in HC compared to OA patients (negative log_2_ FC). Abbreviations: CAR, acylcarnitine; GCA, glycocholic acid; GCDCA, glycochenodeoxycholic acid; GDCA, glycodeoxycholic acid; PE, phosphatidylethanolamine; TCA, taurocholic acid; TDCA, taurodeoxycholic acid. (For interpretation of the references to colour in this figure legend, the reader is referred to the Web version of this article.)

In total, 33 serum metabolites were altered in OA patients compared to HC. Most of these metabolites were present at significantly (q < 0.05) higher concentrations in HC than in individuals with knee OA (Fig. 1 and Supplementary Table S2). The pathway analysis of the significantly (p < 0.05) altered metabolites for serum revealed that pathways involving amino acid synthesis and metabolism were the most prominent (Supplementary Fig. S2 and Supplementary Table S3). These included branched-chain amino acids (isoleucine, leucine, and valine), along with alanine, asparagine, histidine, ornithine, proline, threonine, tryptophan, and tyrosine. Several amino acid derivatives were also found in lower levels in OA patients compared to HC, including 3-hydroxyphenylacetic acid, 4-guanidinobutanoate, creatine, indole-3-acetic acid, methionine sulfoxide, and N-acetylphenylalanine.

Bile acids were notably lower in OA patients compared to HC, specifically glycocholic acid (GCA), glycochenodeoxycholic acid (GCDCA), glycodeoxycholic acid (GDCA), taurocholic acid (TCA), and taurodeoxycholic acid (TDCA). Lower levels of short-chain acylcarnitines (CARs 3:0, 4:0, 5:0), phosphatidylethanolamine (PE) 33:1, cytosine, dihydrouracil, deoxycarnitine, malic acid, trigonelline, and vitamin B7 were also observed in the OA group. In contrast, uracil concentrations were significantly increased in individuals with knee OA compared to HC.

### 3.3 Correlations between serum metabolites and clinical symptoms in OA patients

**Fig. 2:**
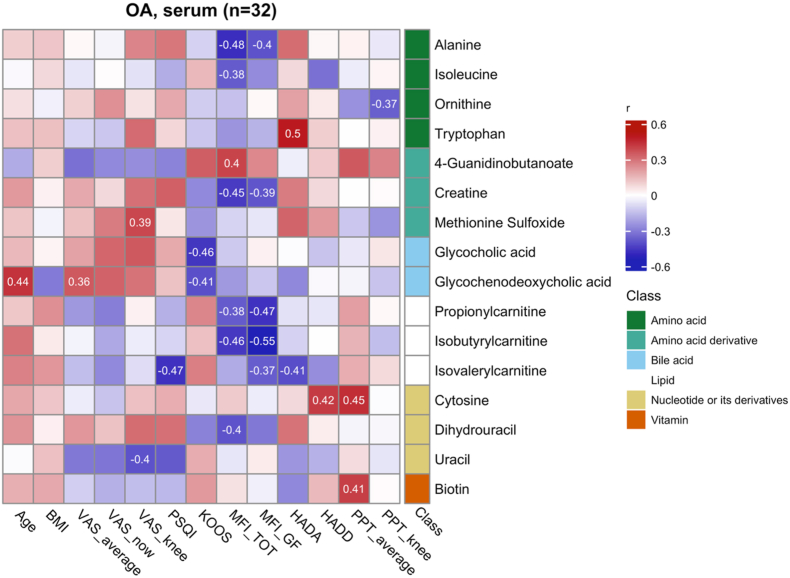
All significantly altered (q < 0.05) serum metabolites between osteoarthritis (OA) patients and healthy controls (HC) with a significant (p < 0.05) correlation with any symptom parameter. The numbers presented are correlation coefficients for the significant correlations, where red indicates a positive correlation, and blue indicates a negative correlation. The metabolites shown were significantly altered compared to HC (q < 0.05) and had a significant (p < 0.05) correlation to pain intensity, sleep disturbances, disability, fatigue, or pressure pain sensitivity. Abbreviations: BMI, body mass index; CAR 3:0, propionylcarnitine; CAR 4:0, isobutyrylcarnitine; CAR 5:0, isovalerylcarnitine; GCA, glycocholic acid; GCDCA, glycochenodeoxycholic acid; HAD-A/D, hospital anxiety and depression scale (anxiety/depression subscales); KOOS, knee injury and osteoarthritis outcome score; MFI TOT, total score from multidimensional fatigue inventory; MFI GF, general fatigue subscale from multidimensional fatigue inventory; PPT, pressure pain threshold; PSQI, Pittsburgh sleep quality index; VAS, visual analogue scale. (For interpretation of the references to colour in this figure legend, the reader is referred to the Web version of this article.)

Among the metabolites differentiating OA patients from HC, Fig. 2 presents the correlations between the serum metabolites and clinical symptoms. Table 2 lists the log_2_ FC and q-values of these metabolites. Reduced levels of amino acids alanine and isoleucine were correlated with greater fatigue score (MFI_TOT). Tryptophan was associated with higher ratings of anxiety (HADA). Ornitine was associated with greater knee pressure pain sensitivity (PPT_knee).

**Table 2:** Presented are the log_2_ fold changes (FC), 95% confidence intervals (CI), and raw and adjusted p-values for the serum and cerebrospinal fluid (CSF) metabolites significantly altered (q < 0.05) compared to controls and with an association to pain intensity, pressure pain sensitivity, fatigue, functional disability, or psychological symptoms in osteoarthritis (OA) patients.

Compound	Abbreviation	Class	Log_2_ FC (95% CI)	p-value	q-value	Matrix
Alanine	Alanine	Amino acid	−0.34 (−0.52–-0.16)	0.00046	0.0026	Serum
Isoleucine	Isoleucine	Amino acid	−0.5 (−0.78–-0.21)	0.00084	0.0043	Serum
Ornithine	Ornithine	Amino acid	−0.59 (−0.9–-0.29)	0.00026	0.0016	Serum
Tryptophan	Tryptophan	Amino acid	−0.42 (−0.68–-0.16)	0.0022	0.0086	Serum
3-Hydroxyphenylacetic acid	3-Hydroxyphenylacetic acid	Amino acid derivative	2.23 (1.82–2.62)	3.7E-17	2.7E-15	CSF
4-Guanidinobutanoate	4-Guanidinobutanoate	Amino acid derivative	−1.94 (−2.49–-1.39)	1.6E-09	8.3E-08	Serum
Creatine	Creatine	Amino acid derivative	−0.25 (−0.45–-0.05)	0.015	0.048	Serum
Histamine	Histamine	Amino acid derivative	1.59 (0.78–2.39)	0.00019	0.0018	CSF
Methionine Sulfoxide	Methionine Sulfoxide	Amino acid derivative	−0.84 (−1.16–-0.52)	2.2E-06	0.000046	Serum
Glycocholic acid	GCA	Bile acid	−1.26 (−1.94–-0.58)	0.00046	0.0026	Serum
Glycochenodeoxycholic acid	GCDCA	Bile acid	−1.7 (−2.39–-1.01)	6.4E-06	0.000085	Serum
Propionylcarnitine	CAR 3:0	Lipid	−0.63 (−0.92–-0.33)	0.000068	0.0006	Serum
Isobutyrylcarnitine	CAR 4:0	Lipid	−0.4 (−0.71–-0.09)	0.013	0.044	Serum
Isovalerylcarnitine	CAR 5:0	Lipid	−0.71 (-1–-0.43)	4.3E-06	0.000065	Serum
Cytosine	Cytosine	Nucleotide or its derivatives	−1.1 (−1.47–-0.73)	1.3E-07	3.3E-06	Serum
Dihydrouracil	Dihydrouracil	Nucleotide or its derivatives	−0.28 (−0.46–-0.1)	0.0028	0.011	Serum
Uracil	Uracil	Nucleotide or its derivatives	0.55 (0.31–0.8)	0.000024	0.00027	Serum
Biotin	Vitamin B7	Vitamin	−0.39 (−0.61–-0.18)	0.00054	0.0029	Serum

Amino acid derivative creatine was negatively correlated with fatigue (MFI), while 4-guanidinobutanoate was positively correlated. Methionine sulfoxide levels positively associated with knee pain intensity (VAS_knee).

The bile acid GCDCA was positively correlated with average pain intensity (VAS_average), even though it was reduced in OA patients. GCA and GCDCA were also associated with worse knee-related symptoms (lower KOOS scores). Among lipids, lower levels of propionylcarnitine (CAR 3:0), isobutyrylcarnitine (CAR 4:0), and isovalerylcarnitine (CAR 5:0) were associated with greater fatigue (negatively correlated with MFI_TOT and MFI_GF). Isovalerylcarnitine (CAR 5:0) was additionally negatively correlated with sleep disturbances (PSQI) and anxiety (HADA).

For nucleotides and their derivatives, cytosine was positively correlated with the average pressure pain threshold (PPT_average), while dihydrouracil was negatively correlated with MFI_TOT. Vitamin B7 was positively correlated with PPT_average. Uracil, which was the only increased serum metabolite in OA patients, was found to be negatively associated with knee pain ratings.

### 3.4 Cerebrospinal fluid metabolite differences between OA patients and NINS controls

**Fig. 3:**
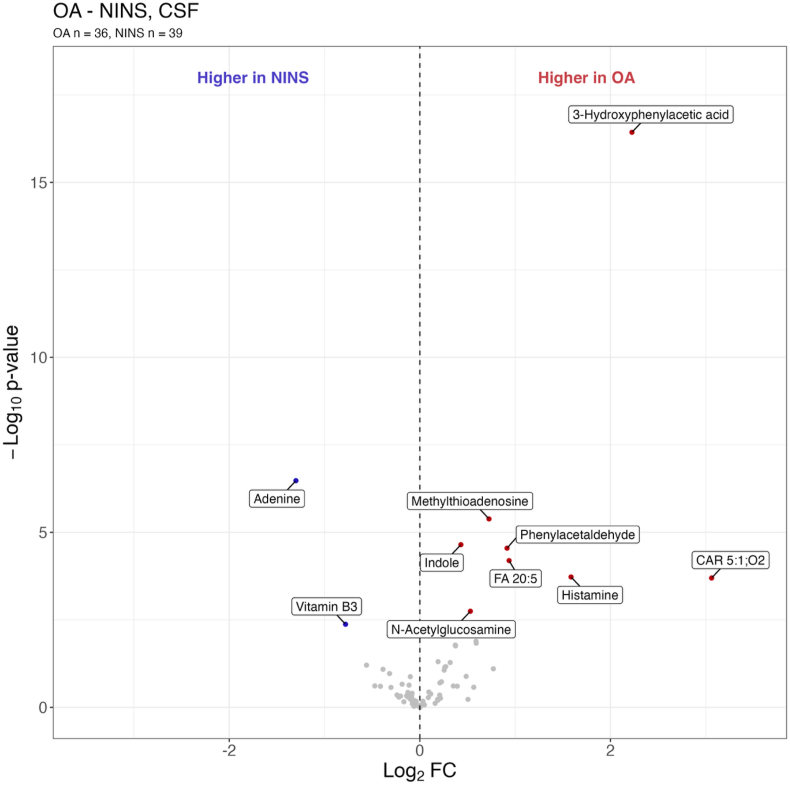
Volcano plot of the cerebrospinal fluid (CSF) metabolites differed between osteoarthritis (OA) patients and control subjects with non-inflammatory neurological symptoms (NINS controls). The x-axis is the log_2_ fold change (FC), and the y-axis is the non-adjusted p-value. The coloured dots have q-values below 0.05. Red dots are the metabolites found in higher concentrations in OA patients compared to NINS controls (positive log_2_ FC). Blue dots are the metabolites found in higher concentrations in NINS compared to OA patients (negative log_2_ FC). Abbreviations: CAR 5:1; O2, Glutarylcarnitine; FA 20:5, Eicosapentaenoic acid. (For interpretation of the references to colour in this figure legend, the reader is referred to the Web version of this article.)

Fig. 3 presents the ten CSF metabolites found significantly (q < 0.05) altered in OA patients compared to NINS controls (Supplementary Table S2). The pathway analysis of the significantly (q < 0.05) altered CSF metabolites revealed that he phenylalanine pathway was altered (Supplementary Fig. S2 and Supplementary Table S3). Knee OA patients exhibited elevated levels of amino acid derivatives 3-hydroxyphenylacetic acid, histamine, and indole. Increased levels were also observed for the lipids CAR 5:1; O2, fatty acid (FA) 20:5, the nucleotide derivative methylthioadenosine, as well as phenylacetaldehyde and N-acetylglucosamine. In contrast, nucleotide adenine and vitamin B3 were significantly higher in NINS controls compared to OA patients.

### 3.5 Correlations between metabolites in CSF and symptoms in OA patients

**Fig. 4:**
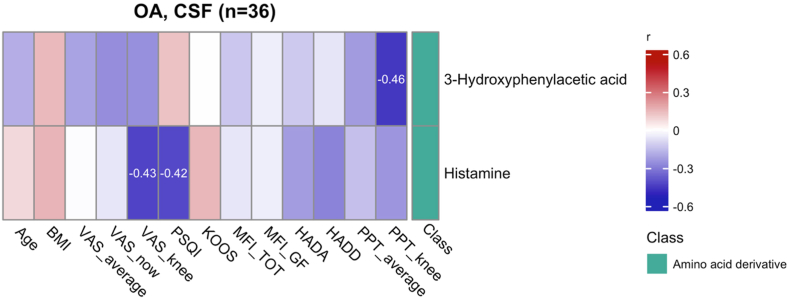
All cerebrospinal fluid (CSF) metabolites significantly (q < 0.05) altered between osteoarthritis (OA) patients and control subjects, with a significant (p < 0.05) correlation with symptoms. The numbers presented are the correlation coefficient for significant correlations (p < 0.05), where red indicates a positive correlation and blue indicates a negative correlation. The metabolites shown were significantly altered (q < 0.05) and had a significant correlation (p < 0.05) to pain intensity, sleep disturbances, disability, fatigue, or pressure pain sensitivity. Abbreviations: BMI, body mass index; HAD-A/D, Hospital Anxiety and Depression Scale (Anxiety/Depression subscales); KOOS, Knee Injury and Osteoarthritis Outcome Score; MFI TOT, total score from Multidimensional Fatigue Inventory; MFI GF, general fatigue subscale from Multidimensional Fatigue Inventory; PPT, Pressure Pain Threshold; PSQI, Pittsburgh Sleep Quality Index; VAS, Visual Analogue Scale. (For interpretation of the references to colour in this figure legend, the reader is referred to the Web version of this article.)

Two CSF metabolites, with elevated levels in individuals with knee OA, were also associated with OA symptoms (Fig. 4). 3-Hydroxyphenylacetic acid was associated with increased knee pressure pain sensitivity (negative association with PPT_knee). Histamine levels were negatively associated with knee pain intensity (VAS_knee) and sleep quality (PSQI). These results indicate that select CSF metabolites are associated with both the presence of knee OA and specific clinical symptom measures.

### 3.6 Correlations of CSF-to-serum ratios and albumin quotient with altered metabolites in knee OA patients

**Fig. 5:**
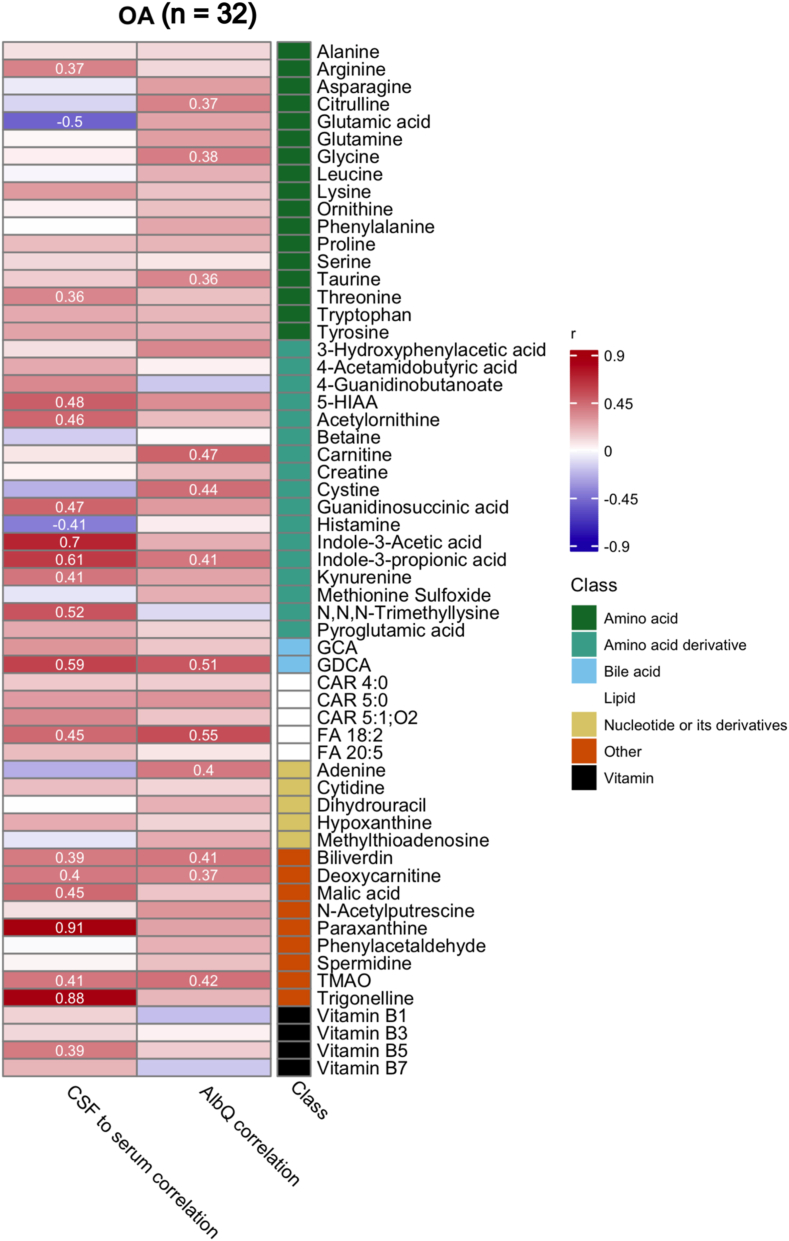
Heatmap showing cerebrospinal fluid (CSF) metabolite correlations in knee osteoarthritis (OA) patients. The left column displays significant (p < 0.05) Spearman's rank correlation coefficients (r) between CSF and serum metabolite levels, and the right column shows correlations between each CSF and serum metabolite ratio and the albumin quotient (AlbQ), a marker of BBB integrity. Metabolites are annotated and sorted by metabolite class. Positive correlations are shown in red, negative in blue, with intensity reflecting correlation strength. Notable metabolites with strong CSF-to-serum correlations include paraxanthine and trigonelle, while several lipids and energy-related compounds in CSF show positive associations with AlbQ, indicating possible blood–brain barrier dysfunction in OA. Abbreviations: 5-HIAA, 5-hydroxyindoleacetic acid; CAR, acylcarnitine; FA, fatty acid; GCA, glycocholic acid; GDCA, glycodeoxycholic acid; TMAO, trimethylamine N-oxide. (For interpretation of the references to colour in this figure legend, the reader is referred to the Web version of this article.)

Fig. 5 shows the association between serum and CSF, and to AlbQ of CSF metabolites in knee OA patients, which revealed several noteworthy correlations between serum and CSF metabolite levels, and some with albumin quotient (AlbQ). Many metabolites showed moderate to strong positive correlations between CSF and serum, suggesting that systemic metabolite levels influence CSF composition. Notably, paraxanthine, trigonelline, and indole-3-acetic acid exhibited high (r ≥ 0.7) CSF-to-serum correlations. In contrast, glutamic acid and histamine showed negative correlations, indicating possible differential regulation or restricted transport across the blood-brain barrier (BBB). Interestingly, some metabolites with significant correlations between CSF and serum levels also correlated with AlbQ, a marker of BBB permeability. Indole-3-propionic acid, GDCA, FA 18:2, biliverdin, deoxycarnitine, and trimethylamine N-oxide (TMAO) had a correlation with both CSF-serum and with AlbQ.

## 4 Discussion

To the best of our knowledge, this is the first study to compare metabolites in CSF and serum between individuals with knee OA and controls while examining their correlation with symptom parameters in OA patients. The serum metabolite analysis revealed differences between individuals with knee OA and HC. OA patients exhibited lower levels of amino acids, bile acids, and lipids, but had significantly elevated uracil concentrations. Correlation analysis showed that bile acid GCDCA, although reduced in OA, was associated with pain and the severity of OA symptoms. In contrast, several amino acids and lipids were negatively associated with fatigue. Additionally, nucleotides and their derivatives displayed correlations with knee pain intensity, pressure pain sensitivity, and fatigue, highlighting potential serum metabolic markers of OA symptoms. Contrary to our findings of reduced concentrations of metabolites in serum, metabolites in the CSF of OA patients were generally higher compared to NINS controls. These amino acid derivatives were related to symptoms, namely histamine, which is negatively associated with knee pain intensity and sleep disturbance, and 3-hydroxyphenlyacetic acid, which is associated with pressure pain sensitivity at the affected knee.

### 4.1 Systemic metabolic changes in knee OA patients and their relation to symptoms

Our serum metabolomic analysis and existing literature reveal that OA patients exhibit complex metabolic alterations. These alterations are associated with disease severity, systemic inflammation, and patient-reported symptoms. Alterations in circulating amino acids, such as branched-chain amino acids (BCAA), histidine, arginine, and tryptophan, have been previously reported in knee OA (Zhai et al., 2010; Zhang et al., 2016a, 2016b; Pascale et al., 2013). The BCAAs share structural similarities, characterized by branched aliphatic side chains, and are essential components of the human diet, accounting for approximately one-third of skeletal muscle protein content. In the present study, BCAA concentrations were reduced in individuals with knee OA compared to healthy serum controls. This finding contrasts with previous research that reported elevated BCAA levels in association with obesity (BMI ≥30 kg/m^2^) and metabolic syndrome (Batch et al., 2013), which are conditions that often co-occur with OA (Schett et al., 2013; Yoshimura et al., 2012). A contributing factor to this finding may be that the patient cohort had been fasting since the previous evening, whereas the healthy controls were not subject to the same fasting conditions. Fasting is known to induce metabolic changes, altering the circulating levels of various metabolites, including amino acids, glucose, and lipids. These changes can confound comparisons between patient and control groups if nutritional status is not matched. For instance, a prior study comparing fasting men and non-fasting women reported differences in metabolite profiles between the two groups. Notably, this study found that only glutamine exhibited a statistically significant change, with increased levels observed in the fasting group (Carayol et al., 2015). Interestingly, this is the opposite trend to what we observed in our cohort, suggesting that fasting alone may not fully explain the metabolite differences identified. Moreover, although the OA group demonstrated a significantly higher mean BMI than the control group (27.8 vs. 24.9), the average BMI of the OA cohort did not fall within the clinical range classified as obesity. Our results suggest that OA may be associated with a distinct systemic metabolic profile differentiating from HC, as the alterations in branched-chain amino acids were prominent in the pathways analysis, potentially shaped by local joint inflammation (Cicuttini and Wluka, 2014).

Significant associations were observed between specific metabolites and patient-reported outcomes. Notably, alanine and isoleucine showed negative associations with measures of fatigue (total score of MFI), suggesting that lower concentrations of these amino acids may be linked to more severe fatigue. Alanine has previously been implicated in subchondral bone sclerosis and appears to contribute to elevated energy demand in osteoarthritic joints (Yang et al., 2016). However, isoleucine exerts a potent hypoglycemic effect by significantly increasing insulin-independent glucose uptake in skeletal muscle, enhancing whole-body glucose oxidation, and inhibiting hepatic gluconeogenesis through downregulating key gluconeogenic enzymes without stimulating insulin secretion (Doi et al., 2007). These findings suggest that disruptions in amino acid metabolism, such as alanine and isoleucine, may contribute to fatigue symptoms in OA through mechanisms related to energy demand and glucose regulation.

A previous study identified distinct patterns of oxidized, nitrated, and glycated amino acids that could enable early detection and classification of arthritis (Ahmed et al., 2016). Our results may expand on this work by showing that specific modified amino acids are also associated with patient-reported symptoms, suggesting their potential relevance to disease severity. Amino acid derivatives 4-guanidinobutanoate and methionine sulfoxide were positively correlated with fatigue and knee pain (VAS_knee), respectively. Previous studies also show that oxidative stress is significantly elevated in osteoarthritis (OA) patients, with higher total peroxide levels and oxidative stress index alongside reduced antioxidant capacity compared to controls (Tootsi et al., 2017). Furthermore, oxidative stress is associated with metabolic disturbances like impaired lipid metabolism and dysglycemia, which correlate with the severity and progression of OA (Tootsi et al., 2020). Within the lipidomic profile, acylcarnitines (CARs) 3:0 and 4:0 were decreased in OA patients and showed negative correlations with fatigue, whereas CAR 5:0 was negatively correlated with sleep disturbances. Acylcarnitines are essential in fatty acid oxidation of the mitochondria, and disruption of this may also point to increased oxidative stress or mitochondrial dysfunction. These findings highlight a potential link between lipid metabolism and key symptoms such as fatigue and disturbed sleep, which are commonly reported by individuals with OA (Hawker et al., 2010). Our study further suggests that increased oxidative stress or amino acid degradation may contribute to the overall symptom burden in knee OA patients.

Our findings revealed associations between specific circulating bile acids and clinical outcomes in knee OA, reinforcing and extending emerging evidence of a gut-joint axis mediated by bile acid signaling (Yang et al., 2025). In line with the recent study demonstrating the protective effects of the conjugated secondary bile acid glycoursodeoxycholic acid (GUDCA) via farnesoid-X-receptor (FXR) inhibition and glucagon-like peptide-1 (GLP-1) signaling in experimental OA models (Yang et al., 2025), we observed that the conjugated primary bile acids, glycocholic acid (GCA) and glycochenodeoxycholic acid (GCDCA), were associated with potentially beneficial effects in knee OA patients. Specifically, both GCA and GCDCA exhibited negative associations with KOOS scores and anxiety and depression as measured by the HADS scale, suggesting they may have a potential protective role in both disease severity and psychological symptoms. In contrast, GCDCA was positively associated with average pain intensity and age. Further research is needed to clarify this association's nature and determine whether age-related changes in bile acid metabolism are linked to pain severity in older adults with knee OA. Taken together, our results support a multifactorial role for bile acids in OA, consistent with the concept of a gut-joint axis. Given the availability of FDA-approved drugs that modulate bile acid metabolism (e.g., UDCA, FXR agonists/antagonists, and GLP-1R agonists), these insights may inform future personalized treatment strategies for OA that integrate metabolic and microbiota-focused approaches (Yang et al., 2025).

DNA methylation is catalyzed by DNMTs using S-adenosyl methionine, adding a methyl group to cytosine to form 5-methylcytosine (5 mC) (Cai et al., 2022). Methylation in promoter regions typically results in suppression of gene transcription (Cai et al., 2022). In our study, lower cytosine levels were associated with decreased pressure pain sensitivity, suggesting a link between methylation-related metabolites and pain sensitivity. Additionally, lower levels of dihydrouracil and uracil were associated with increased fatigue and knee pain, highlighting a potential role for nucleotide metabolism (Caldo et al., 2024) in modulating osteoarthritis symptoms. These findings suggest that systemic metabolic alterations, particularly in amino acids, bile acids, and nucleotides, may contribute to pain and fatigue symptoms in OA patients.

### 4.2 Metabolic changes in the CSF of knee OA patients and their relation to symptoms

The CSF analysis revealed several metabolites altered between OA patients and NINS controls. Two of the elevated substances in OA patients were also associated with symptom severity, namely 3-HPAA and histamine. We are unaware of any previously reported links between pain and the gut-derived 3-HPAA (Del Bo et al., 2019; Dias et al., 2022), nor did we see an altered concentration in serum in our cohort. Furthermore, no correlations were found between serum and CSF levels. Hence, we can only establish that in our OA patients, elevated levels of 3-HPAA in CSF were associated with increased pressure pain sensitivity around the affected knee, which would translate to tenderness in clinical terms.

In patients with OA, CSF histamine concentrations were inversely correlated with pain severity and disrupted sleep patterns. While pain levels remained elevated overall, this pattern may suggest a potential modulatory role of central histamine in these symptoms. This interpretation aligns with histamine's established functions as a neurotransmitter and immune modulator involved in pain processing, inflammatory responses, and the regulation of wakefulness (Haas et al., 2008; Panula and Nuutinen, 2013; Panula et al., 2015; Pini et al., 2016). The inverse relationship between CSF histamine and pain severity may reflect alterations in central histaminergic signaling pathways during chronic pain states, potentially driven by neuroinflammation or dysfunction of histamine-producing neurons (Khalilzadeh et al., 2018; Smith et al., 2007). Alternatively, elevated central histamine levels could represent an adaptive or compensatory mechanism to attenuate nociceptive signaling and inflammatory cascades, thereby limiting pain persistence. Conversely, reductions in CSF histamine might facilitate ongoing pain by weakening these modulatory effects (Erfanparast et al., 2010; Tamaddonfard and Hamzeh-Gooshchi, 2014). This central histamine-mediated antinociception aligns with experimental evidence demonstrating histamine's pain-attenuating effects within the brain, contrasting its peripheral nociceptive role (Panula and Nuutinen, 2013).

Furthermore, the observed inverse correlation between histamine concentrations in the CSF and serum compartments indicates distinct regulatory mechanisms governing histamine distribution across the BBB. Moreover, the lack of association with BBB permeability (AlbQ) underscores its predominant synthesis and release within the CNS, highlighting histamine's role as an intrinsic neurotransmitter rather than a peripheral mediator (Lindskog, 2017; Parsons and Ganellin, 2006). These findings suggest that central and peripheral histamine pools may be differentially regulated. However, given the cross-sectional design, no causal inferences can be made, and longitudinal studies are needed to clarify potential implications for pain and sleep disturbances.

### 4.3 Associations between serum-CSF crosstalk and BBB permeability

Several metabolites demonstrated a positive correlation between CSF and serum concentrations in individuals with knee OA, indicating a possible metabolic connection between the peripheral and central compartments. Six metabolites (indole-3-propionic acid, GDCA, FA 18:2, biliverdin, deoxycarnitine, and TMAO) exhibited significant weak to moderate CSF-serum correlations, and their CSF levels were associated with BBB permeability, indicating that even subclinical alterations of BBB integrity may be relevant. However, none of these metabolites were significantly associated with clinical symptom measures, such as pain, fatigue, mood, or sleep quality. Further investigation is needed to clarify their potential relevance to disease mechanisms or progression as well as to establish if changes in BBB permeability are present in OA.

### 4.4 Strengths and limitations

This study had several strengths. It incorporated detailed symptom measures, including pain, fatigue, mood, and sleep quality, and explored systemic and potential central mechanisms. In addition, the nearly simultaneous sampling time of serum and CSF in the patient group and parallel analysis were significant assets when comparing serum and CSF levels and albumin quotas. However, there are also limitations. This study reports associations, not causal relationships, between metabolites and clinical outcomes. The generally small to moderate correlations are consistent with complex biological systems and warrant validation in larger, longitudinal cohorts. Although samples were collected uniformly across OA patients and healthy controls, diurnal variation may affect them. OA patients were fasting overnight, while the control groups were not, which may impact some group difference analysis. In addition, the premedication given to OA patients before surgery could influence the results.

Finally, as we were not granted ethical permission for lumbar punctures in healthy subjects, we had to rely on NINS as non-healthy CSF controls. Although these patients were not diagnosed with an inflammatory neurological condition, and the majority did not suffer from any painful condition, they cannot be considered healthy and were on average younger. Hence, the reported data regarding group differences in CSF metabolites must be regarded as preliminary and need to be replicated in comparison with a healthy control group.

### 4.5 Conclusions

This study is among the first to characterize metabolic alterations in both serum and CSF from human knee OA patients, linking these changes to symptoms. Unlike most preclinical research, our human data reveal systemic metabolic differences, such as reduced branched-chain amino acids and bile acids, and elevated CSF metabolites, including histamine and 3-hydroxyphenylacetic acid, which correlate with pain, fatigue, and sleep disturbances. The observed relationships between serum and CSF metabolites and blood-brain barrier permeability suggest that even subclinical BBB changes may affect CSF metabolites. Our integrated serum–CSF approach, combined with detailed symptom assessment, provides insight into metabolic and neurochemical correlates of symptoms in OA. However, given the cross-sectional design, these associations cannot be considered specific to OA or indicative of causality. Nevertheless, the findings may help inform future studies exploring candidate biomarkers and their potential relevance for personalized treatment strategies.

## CRediT authorship contribution statement

**Aline U. Bjerkhaug:** Writing – review & editing, Writing – original draft, Visualization, Validation. **Jenny E. Jakobsson:** Writing – review & editing, Writing – original draft, Formal analysis, Data curation. **Aisha S. Ahmed:** Writing – review & editing, Funding acquisition, Data curation. **Henrik Carlsson:** Writing – review & editing, Methodology, Investigation. **Ida Erngren:** Writing – review & editing, Methodology, Investigation. **Asma Al-Grety:** Writing – review & editing, Methodology, Investigation. **Alex Bersellini Farinotti:** Writing – review & editing, Methodology, Investigation. **Camilla I. Svensson:** Writing – review & editing, Methodology, Funding acquisition. **Kim Kultima:** Writing – review & editing, Supervision, Methodology, Investigation, Funding acquisition, Conceptualization. **Eva Kosek:** Writing – review & editing, Supervision, Methodology, Investigation, Funding acquisition, Conceptualization.

## Disclosure

The authors declare no competing interests.

During the preparation of this work the author*s* used Grammarly and ChatGPT (OpenAI) in order to improve readability and language. After using this tool/service, the author*s* reviewed and edited the content as needed and take*s* full responsibility for the content of the publication.

## Funding

This work was supported by Stockholm County Council, 10.13039/501100004359Swedish Research Council (K2013-52X-22199-01-3 for EK and 542-2013-8373 for CIS), Knut and Alice Wallenberg Foundation (CIS), 10.13039/501100007949Swedish Rheumatism Association (ASA), King Gustav V Foundation Sweden (ASA), 10.13039/501100014688Region Uppsala (ALF-grant (EK)), 10.13039/501100014688Region Uppsala (ALF-grant and R&D funds (KK)), Stockholm County Council (ALF-grant (EK)), Magnus Bergvall's Foundation (KK), and Eli Lilly. The research was also funded by the 10.13039/501100004963European Union Seventh Framework Programme (FP7/2007-2013), under grant agreement no. 602919, and a generous donation from the Lundblad family. The funding sources had no influence on study design or scientific content of this manuscript.

## Declaration of competing interest

The authors declare that they have no known competing financial interests or personal relationships that could have appeared to influence the work reported in this paper.

## Data Availability

Data will be made available on request.
