# Supplementary material for: Distinct serum and cerebrospinal fluid metabolic signatures associate with pain and fatigue in knee osteoarthritis
Source: Brain Behav Immun Health. 2026 Mar 27;53:101227. doi: 10.1016/j.bbih.2026.101227 (PMC13066801; doi:10.1016/j.bbih.2026.101227)
Supplement: Multimedia component 1 [file mmc1.docx]

Supplementary material

# Supplementary Figures

Supplementary Figure S1. Principal component analysis (PCA) was performed on the two different methods, reverse-phase liquid chromatography (RPLC; A-B) and hydrophilic liquid chromatography (HILIC; C-D) and for serum/cerebrospinal fluid (CSF) respectively.


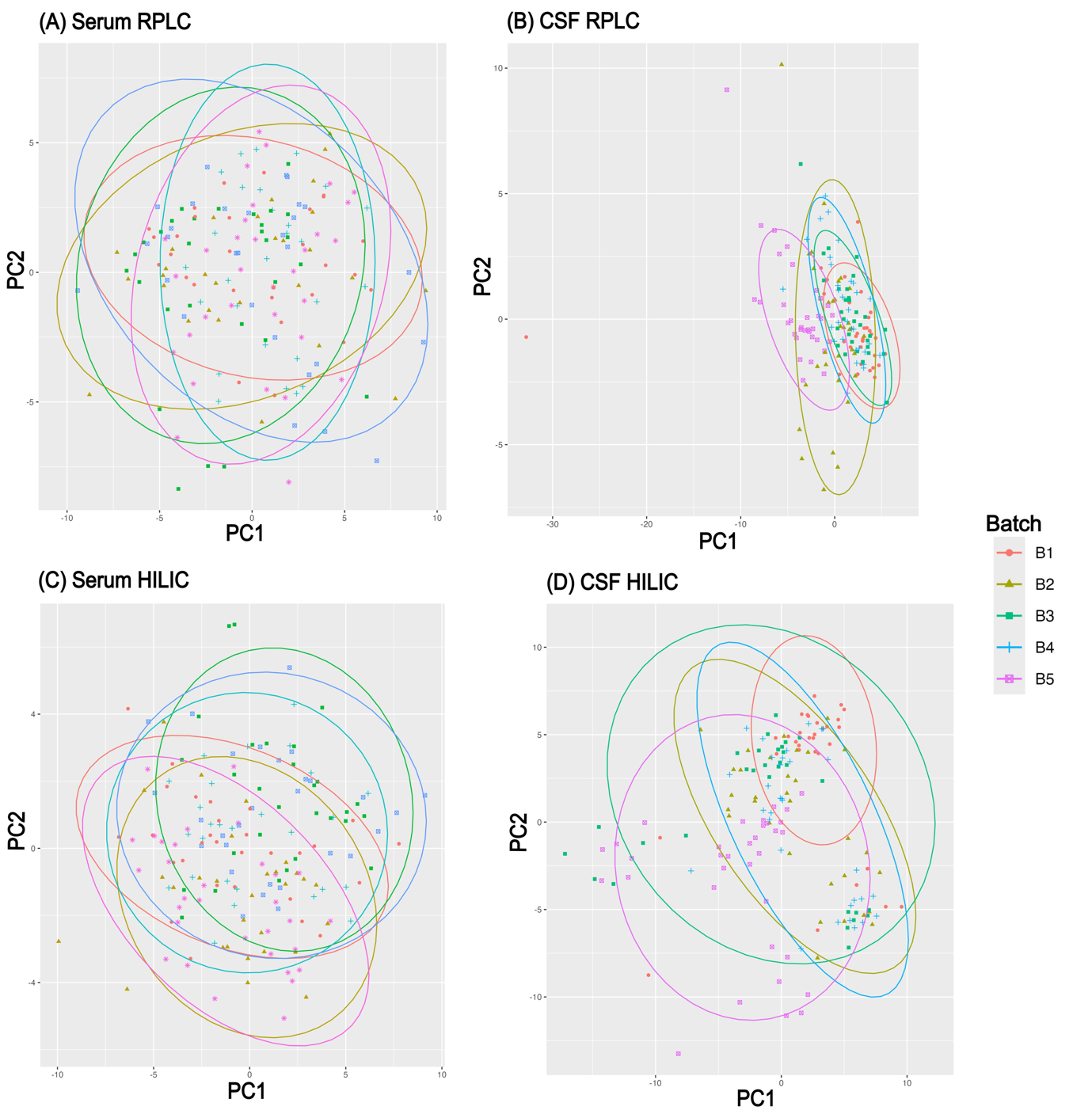


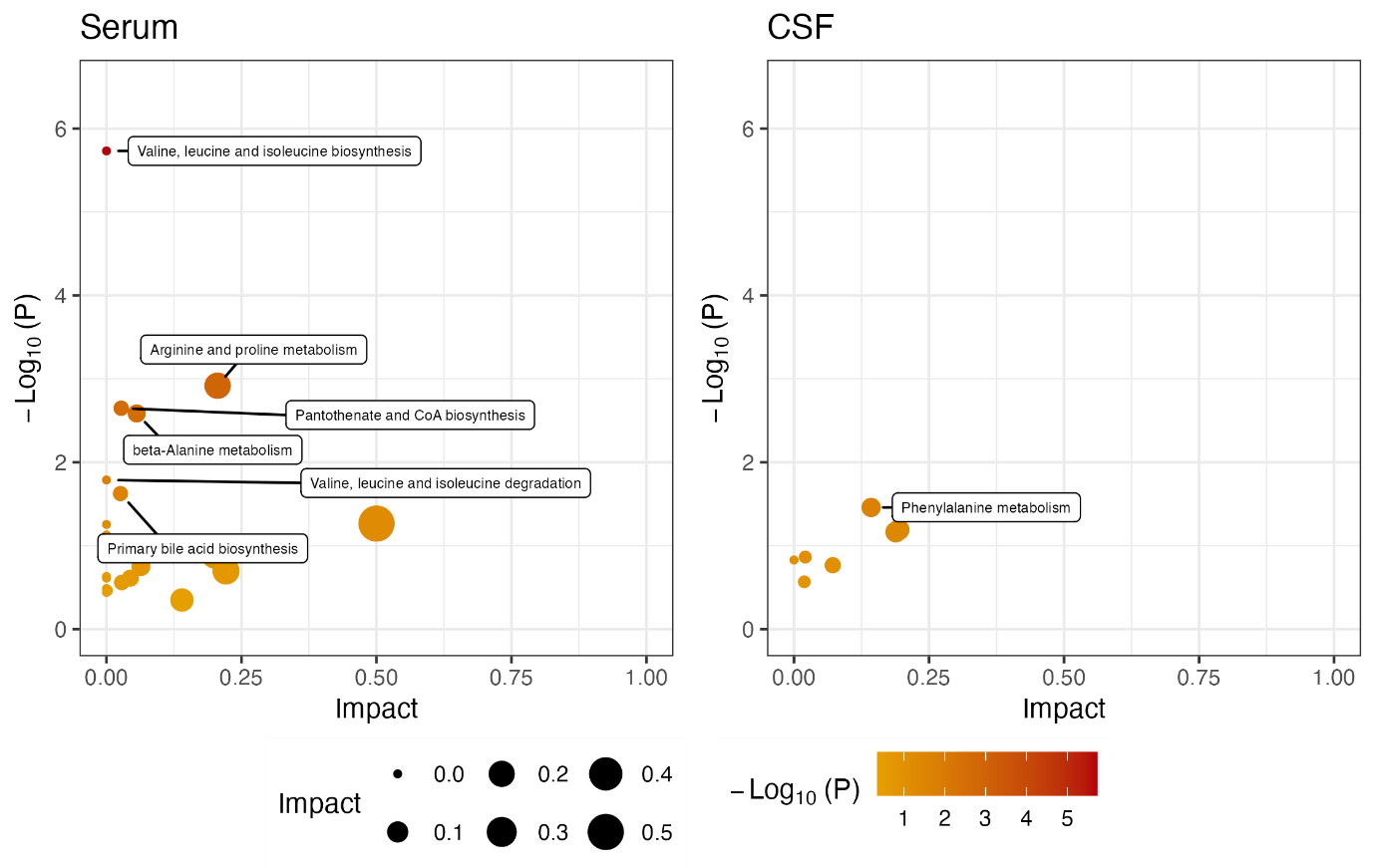


Supplementary Figure S2. Overview of altered metabolic pathways in serum and CSF in OA patients. The pathway analysis was performed using the online tool Metaboanalyst for serum and cerebrospinal fluid (CSF) metabolites, respectively.

# S**upplementary Tables**

Supplementary Table S1. The table lists the targeted metabolites in serum, cerebrospinal fluid (CSF), or both. For each metabolite, the corresponding liquid chromatography-mass spectrometry (LC-MS) acquisition method, and coefficient of variance (CV) for quality controls samples are provided. The LC-MS methods used were based on reverse-phase liquid chromatography (RPLC) or hydrophilic interaction liquid chromatography (HILIC). The metabolites are grouped by metabolite class.

| **Compound** | **Abbreviation** | **Class** | **Method** | **Matrix** | **CV (%)** | |
| --- | --- | --- | --- | --- | --- | --- |
|  |  |  |  |  | **Serum** | **CSF** |
| Alanine | Alanine | Amino acid | HILIC | Serum and CSF | 9.23 | 12.5 |
| Arginine | Arginine | Amino acid | HILIC | Serum and CSF | 13.5 | 15.46 |
| Asparagine | Asparagine | Amino acid | HILIC | Serum and CSF | 13.24 | 18.81 |
| Citrulline | Citrulline | Amino acid | HILIC | Serum and CSF | 20.79 | 24.16 |
| Glutamic acid | Glutamic acid | Amino acid | HILIC | Serum and CSF | 13.75 | 23.46 |
| Glutamine | Glutamine | Amino acid | HILIC | Serum and CSF | 11.25 | 14.88 |
| Glycine | Glycine | Amino acid | HILIC | Serum and CSF | 13.87 | 20.7 |
| Histidine | Histidine | Amino acid | RPLC | Serum | 3.56 |  |
| Homocysteine | Homocysteine | Amino acid | RPLC | Serum | 18 |  |
| Isoleucine | Isoleucine | Amino acid | HILIC | Serum | 11.81 |  |
| Leucine | Leucine | Amino acid | HILIC | Serum and CSF | 13.45 | 33.48 |
| Lysine | Lysine | Amino acid | HILIC | Serum and CSF | 13.47 | 19.33 |
| Ornithine | Ornithine | Amino acid | HILIC | Serum and CSF | 17.96 | 19.78 |
| Phenylalanine | Phenylalanine | Amino acid | HILIC | Serum and CSF | 14.02 | 29.53 |
| Proline | Proline | Amino acid | HILIC | Serum and CSF | 7.91 | 16.02 |
| Serine | Serine | Amino acid | HILIC | Serum and CSF | 11.57 | 16.61 |
| Taurine | Taurine | Amino acid | HILIC | Serum and CSF | 11.41 | 15.53 |
| Threonine | Threonine | Amino acid | HILIC | Serum and CSF | 14.62 | 21.77 |
| Tryptophan | Tryptophan | Amino acid | HILIC | Serum and CSF | 14.12 | 27.23 |
| Tyrosine | Tyrosine | Amino acid | RPLC | Serum and CSF | 24.04 | 24.71 |
| Valine | Valine | Amino acid | HILIC | Serum | 8.01 |  |
| 3-Hydroxyanthranilic acid | 3-Hydroxyanthranilic acid | Amino acid derivative | HILIC | CSF |  | 17.51 |
| 3-Hydroxyphenylacetic acid | 3-Hydroxyphenylacetic acid | Amino acid derivative | HILIC | Serum and CSF | 9.71 | 13.38 |
| 3-Methoxytyrosine | 3-Methoxytyrosine | Amino acid derivative | RPLC | Serum | 20.31 |  |
| 3-Methylindole | 3-Methylindole | Amino acid derivative | HILIC | CSF |  | 12.5 |
| 4-Acetamidobutyric acid | 4-Acetamidobutyric acid | Amino acid derivative | HILIC | Serum and CSF | 18.75 | 19.28 |
| 4-Guanidinobutanoate | 4-Guanidinobutanoate | Amino acid derivative | RPLC | Serum and CSF | 22.86 | 19.26 |
| 4-Hydroxyproline | 4-Hydroxyproline | Amino acid derivative | HILIC | Serum | 12.23 |  |
| 5-Hydroxyindole-3-acetic acid | 5-HIAA | Amino acid derivative | HILIC | Serum and CSF | 14.55 | 28.88 |
| 5-Hydroxytryptophan | 5-Hydroxytryptophan | Amino acid derivative | RPLC | Serum | 25.37 |  |
| Acetylornithine | Acetylornithine | Amino acid derivative | HILIC and RPLC | Serum and CSF | 18.15 | 27.55 |
| Asymmetric dimethylarginine | ADMA | Amino acid derivative | HILIC | Serum | 31.64 |  |
| Betaine | Betaine | Amino acid derivative | HILIC | Serum and CSF | 10.79 | 10.99 |
| Carnitine | Carnitine | Amino acid derivative | HILIC | Serum and CSF | 9 | 15.75 |
| Creatine | Creatine | Amino acid derivative | HILIC | Serum and CSF | 8.97 | 14.53 |
| Creatinine | Creatinine | Amino acid derivative | HILIC | Serum | 9.15 |  |
| Cystine | Cystine | Amino acid derivative | HILIC | Serum and CSF | 12.18 | 21.71 |
| Guanidinoacetic acid | Guanidinoacetic acid | Amino acid derivative | HILIC | Serum | 13.36 |  |
| Guanidinosuccinic acid | Guanidinosuccinic acid | Amino acid derivative | HILIC | Serum and CSF | 18.86 | 25.72 |
| Hippuric acid | Hippuric acid | Amino acid derivative | RPLC | Serum | 11.92 |  |
| Histamine | Histamine | Amino acid derivative | HILIC | Serum and CSF | 10.66 | 25.55 |
| Indole | Indole | Amino acid derivative | HILIC | CSF |  | 16.85 |
| Indole-3-acetic acid | Indole-3-acetic acid | Amino acid derivative | RPLC | Serum and CSF | 6.54 | 12.84 |
| Indole-3-methylacetic acid | Indole-3-methylacetic acid | Amino acid derivative | RPLC | Serum | 32.45 |  |
| indole-3-propionic acid | Indole-3-propionic acid | Amino acid derivative | RPLC | Serum and CSF | 7.4 | 22.27 |
| Kynurenic acid | Kynurenic acid | Amino acid derivative | HILIC | Serum | 21.43 |  |
| Kynurenine | Kynurenine | Amino acid derivative | HILIC | Serum and CSF | 14.81 | 37.08 |
| Methionine sulfoxide | Methionine sulfoxide | Amino acid derivative | HILIC | Serum and CSF | 19.01 | 20.35 |
| Methylguanidine | Methylguanidine | Amino acid derivative | HILIC | CSF |  | 20.42 |
| Methylhistidin | Methylhistidin | Amino acid derivative | RPLC | Serum | 6.82 |  |
| N-Acetylalanine | N-Acetylalanine | Amino acid derivative | RPLC | Serum | 18.63 |  |
| N-Acetylglycine | N-Acetylglycine | Amino acid derivative | RPLC | Serum | 23.22 |  |
| N-Acetylphenylalanine | N-Acetylphenylalanine | Amino acid derivative | RPLC | Serum | 10.63 |  |
| N-Acetyltryptophan | N-Acetyltryptophan | Amino acid derivative | RPLC | Serum | 19.83 |  |
| N,N,N-Trimethyllysine | N,N,N-Trimethyllysine | Amino acid derivative | RPLC | Serum and CSF | 3.93 | 25.53 |
| Pyroglutamic acid | Pyroglutamic acid | Amino acid derivative | RPLC | Serum and CSF | 14.75 | 13.98 |
| Quinolinic acid | Quinolinic acid | Amino acid derivative | RPLC | Serum | 26.74 |  |
| Serotonin | Serotonin | Amino acid derivative | HILIC | Serum | 18.37 |  |
| Glycocholic acid | GCA | Bile acid | RPLC | Serum and CSF | 7.66 | 24.79 |
| Glycochenodeoxycholic acid | GCDCA | Bile acid | RPLC | Serum | 7.24 |  |
| Glycodeoxycholic acid | GDCA | Bile acid | RPLC | Serum and CSF | 7.67 | 24.92 |
| Glycohyocholic acid | GHCA | Bile acid | RPLC | Serum | 10.74 |  |
| Glycohyodeoxycholic acid | GHDCA | Bile acid | RPLC | Serum | 20.55 |  |
| Taurocholic acid | TCA | Bile acid | RPLC | Serum | 14.23 |  |
| Taurodeoxycholic acid | TDCA | Bile acid | RPLC | Serum | 12.32 |  |
| Acetylcarnitine | CAR 2:0 | Lipid | HILIC | Serum | 21.17 |  |
| Propionylcarnitine | CAR 3:0 | Lipid | HILIC | Serum | 9.35 |  |
| Isobutyrylcarnitine | CAR 4:0 | Lipid | HILIC and RPLC | Serum and CSF | 10.9 |  |
| Isovalerylcarnitine | CAR 5:0 | Lipid | HILIC | Serum and CSF | 9.72 |  |
| Glutarylcarnitine | CAR 5:1;O2 | Lipid | HILIC | Serum and CSF | 10.67 | 17.89 |
| Hexanoylcarnitine | CAR 6:0 | Lipid | HILIC | Serum | 9.68 | 17.89 |
| Octanoylcarnitine | CAR 8:0 | Lipid | HILIC | Serum | 9.92 | 12.54 |
| Decanoylcarnitine | CAR 10:0 | Lipid | HILIC | Serum | 8.97 | 18.75 |
| Lauroylcarnitine | CAR 12:0 | Lipid | HILIC | Serum | 11.85 |  |
| Palmitoylcarnitine | CAR 16:0 | Lipid | HILIC | Serum | 17 |  |
| Ethanolamine | Ethanolamine | Lipid | HILIC | CSF |  | 21.8 |
| Linoleic acid | FA 18:2 | Lipid | HILIC | Serum and CSF | 9.93 | 18.11 |
| Eicosapentaenoic acid | FA 20:5 | Lipid | HILIC | Serum and CSF | 11.76 | 27.69 |
| PE 15:0-18:1 | PE 33:1 | Lipid | RPLC | Serum | 22.54 |  |
| Sphingomyelin | SM 36:1;O2 | Lipid | HILIC | Serum | 46.02 |  |
| 4-Pyridoxic acid | 4-Pyridoxic acid | Nucleotide or its derivatives | RPLC | Serum | 18.35 |  |
| Adenine | Adenine | Nucleotide or its derivatives | HILIC | Serum and CSF | 32.68 | 15.8 |
| Cytidine | Cytidine | Nucleotide or its derivatives | HILIC | Serum and CSF | 25.07 | 15.18 |
| Cytosine | Cytosine | Nucleotide or its derivatives | HILIC | Serum | 11.46 |  |
| Dihydrouracil | Dihydrouracil | Nucleotide or its derivatives | HILIC | Serum and CSF | 9.3 | 13.78 |
| Guanosine | Guanosine | Nucleotide or its derivatives | HILIC | CSF |  | 42.52 |
| Hypoxanthine | Hypoxanthine | Nucleotide or its derivatives | HILIC | Serum and CSF | 8.8 | 23.37 |
| Inosine | Inosine | Nucleotide or its derivatives | HILIC | CSF |  | 26.41 |
| Methylthioadenosine | Methylthioadenosine | Nucleotide or its derivatives | RPLC | Serum and CSF | 20.84 | 22.01 |
| Orotic acid | Orotic acid | Nucleotide or its derivatives | HILIC | CSF |  | 22.79 |
| Thymine | Thymine | Nucleotide or its derivatives | HILIC | Serum | 14.47 |  |
| Uracil | Uracil | Nucleotide or its derivatives | RPLC | Serum | 17.32 |  |
| Uric acid | Uric acid | Nucleotide or its derivatives | RPLC | CSF |  | 12.45 |
| Xanthine | Xanthine | Nucleotide or its derivatives | RPLC | Serum | 6.19 |  |
| 2-Deoxy-D-glucose | 2-Deoxy-D-glucose | Other | RPLC | Serum | 36.29 |  |
| 3-Amino-4-hydroxybenzoic acid | 3-Amino-4-hydroxybenzoic acid | Other | HILIC | Serum | 9.41 |  |
| Biliverdin | Biliverdin | Other | RPLC | Serum and CSF | 18.2 | 24.55 |
| Cadaverine | Cadaverine | Other | HILIC | Serum | 5.14 |  |
| Carnosine | Carnosine | Other | HILIC | Serum | 20.77 |  |
| Choline | Choline | Other | HILIC | CSF |  | 13.44 |
| Citric acid | Citric acid | Other | RPLC | CSF |  | 15.18 |
| Deoxycarnitine | Deoxycarnitine | Other | HILIC | Serum and CSF | 9.35 | 15.32 |
| Hypotaurine | Hypotaurine | Other | HILIC | Serum | 34.83 |  |
| Malic acid | Malic acid | Other | RPLC | Serum and CSF | 10.46 | 17.51 |
| N-Acetylputrescine | N-Acetylputrescine | Other | HILIC | Serum and CSF | 10.08 | 23.82 |
| Paraxanthine | Paraxanthine | Other | RPLC | Serum and CSF | 5.65 | 22.02 |
| Phenylacetaldehyde | Phenylacetaldehyde | Other | HILIC | Serum and CSF | 8.26 | 13.02 |
| Phosphonoacetate | Phosphonoacetate | Other | RPLC | Serum | 17.37 |  |
| Spermidine | Spermidine | Other | RPLC | Serum and CSF | 8.44 | 21.88 |
| Trimethylamine-N-oxide | TMAO | Other | HILIC | Serum and CSF | 11.6 | 14.3 |
| Trigonelline | Trigonelline | Other | HILIC | Serum and CSF | 16.93 | 37.66 |
| Hexose (sum of hexoses) | Hexose | Sugar or its derivatives | RPLC | CSF |  | 18.25 |
| N-Acetylglucosamine | N-Acetylglucosamine | Sugar or its derivatives | HILIC | CSF |  | 30.73 |
| N-Acetylneuraminate | N-Acetylneuraminate | Sugar or its derivatives | RPLC | CSF |  | 19.68 |
| Riboflavin | Riboflavin | Vitamin | RPLC | Serum | 33.43 |  |
| Retinol | Vitamin A | Vitamin | HILIC | Serum | 16.03 |  |
| Thiamine | Vitamin B1 | Vitamin | HILIC | Serum and CSF | 30.69 | 28.1 |
| Niacinamide | Vitamin B3 | Vitamin | HILIC | Serum and CSF | 9.5 | 15.6 |
| Pantothenic acid | Vitamin B5 | Vitamin | RPLC | Serum and CSF | 24.55 | 22.45 |
| Biotin | Vitamin B7 | Vitamin | HILIC and RPLC | Serum and CSF | 8.91 | 22.05 |

Supplementary Table S2. A total of 120 metabolites were measured in serum, cerebrospinal fluid (CSF), or both. Group comparisons were performed using linear regression to assess differences between patients with osteoarthritis (OA), healthy serum controls (HC), or non-healthy CSF controls with non-inflammatory neurological symptoms (NINS). P-values were adjusted using the Benjamini-Hochberg procedure to control the false discovery rate (FDR). Metabolites with FDR-adjusted q-values<0.05 were considered significant. Empty rows indicate metabolites that were either not detected or did not pass the quality control check.

| **Compound** | **Class** | **Serum** | | | **CSF** | | |
| --- | --- | --- | --- | --- | --- | --- | --- |
|  |  | **Log_2_ FC (95% CI)** | **p-value** | **q-value** | **Log_2_ FC (95% CI)** | **p-value** | **q-value** |
| Alanine | Amino acid | -0.34 (-0.52–-0.16) | 0.00046 | 0.0026 | -0.08 (-0.27–0.11) | 0.4 | 0.76 |
| Arginine | Amino acid | -0.21 (-0.4–-0.03) | 0.024 | 0.067 | -0.13 (-0.41–0.16) | 0.37 | 0.74 |
| Asparagine | Amino acid | -0.34 (-0.54–-0.13) | 0.0016 | 0.0071 | -0.12 (-0.47–0.24) | 0.52 | 0.8 |
| Citrulline | Amino acid | -0.07 (-0.42–0.28) | 0.69 | 0.79 | -0.08 (-0.69–0.53) | 0.8 | 0.88 |
| Glutamate | Amino acid | 0.12 (-0.18–0.42) | 0.43 | 0.57 | -0.17 (-1.01–0.68) | 0.69 | 0.84 |
| Glutamine | Amino acid | -0.14 (-0.32–0.04) | 0.12 | 0.21 | -0.06 (-0.29–0.18) | 0.65 | 0.84 |
| Glycine | Amino acid | -0.12 (-0.4–0.17) | 0.43 | 0.57 | -0.07 (-0.45–0.31) | 0.71 | 0.84 |
| Histidine | Amino acid | -0.25 (-0.36–-0.14) | 2.9e-05 | 0.00028 |  |  |  |
| Homocysteine | Amino acid | -0.01 (-0.5–0.48) | 0.97 | 0.98 |  |  |  |
| Isoleucine | Amino acid | -0.5 (-0.78–-0.21) | 0.00084 | 0.0043 |  |  |  |
| Leucine | Amino acid | -0.38 (-0.61–-0.15) | 0.0018 | 0.0074 | 0.5 (-1.36–2.37) | 0.59 | 0.8 |
| Lysine | Amino acid | -0.25 (-0.48–-0.01) | 0.038 | 0.091 | -0.08 (-0.5–0.34) | 0.71 | 0.84 |
| Ornithine | Amino acid | -0.59 (-0.9–-0.29) | 0.00026 | 0.0016 | -0.3 (-0.85–0.24) | 0.27 | 0.56 |
| Phenylalanine | Amino acid | -0.23 (-0.45–0) | 0.051 | 0.11 | 0 (-0.28–0.29) | 0.98 | 0.99 |
| Proline | Amino acid | -0.73 (-0.96–-0.49) | 4.9e-08 | 1.7e-06 | -0.39 (-0.82–0.05) | 0.082 | 0.28 |
| Serine | Amino acid | -0.08 (-0.27–0.11) | 0.39 | 0.53 | 0.09 (-0.19–0.36) | 0.52 | 0.8 |
| Taurine | Amino acid | -0.04 (-0.24–0.15) | 0.65 | 0.76 | -0.03 (-0.34–0.29) | 0.87 | 0.93 |
| Threonine | Amino acid | -0.44 (-0.71–-0.17) | 0.0016 | 0.0071 | -0.13 (-0.5–0.25) | 0.51 | 0.8 |
| Tryptophan | Amino acid | -0.42 (-0.68–-0.16) | 0.0022 | 0.0086 | 0.27 (-0.02–0.56) | 0.068 | 0.27 |
| Tyrosine | Amino acid | -0.52 (-0.87–-0.18) | 0.0037 | 0.014 | 0.37 (0.07–0.67) | 0.017 | 0.091 |
| Valine | Amino acid | -0.65 (-0.8–-0.49) | 9.5e-12 | 1e-09 |  |  |  |
| 3-Hydroxyanthranilic acid | Amino acid derivative |  |  |  | -0.04 (-0.5–0.43) | 0.88 | 0.93 |
| 3-Hydroxyphenylacetic acid | Amino acid derivative | -0.36 (-0.54–-0.17) | 0.00024 | 0.0016 | 2.23 (1.83–2.63) | 3.7e-17 | 2.7e-15 |
| 3-Methoxytyrosine | Amino acid derivative | -0.15 (-0.35–0.05) | 0.13 | 0.22 |  |  |  |
| 3-Methylindole | Amino acid derivative |  |  |  | 0.03 (-0.18–0.24) | 0.76 | 0.87 |
| 4-Acetamidobutyric acid | Amino acid derivative | -0.31 (-0.8–0.17) | 0.2 | 0.3 | 0.11 (-0.16–0.39) | 0.42 | 0.78 |
| 4-Guanidinobutanoate | Amino acid derivative | -1.94 (-2.49–-1.39) | 1.6e-09 | 8.3e-08 | -0.09 (-0.41–0.23) | 0.58 | 0.8 |
| 4-Hydroxyproline | Amino acid derivative | -0.31 (-0.65–0.03) | 0.075 | 0.15 |  |  |  |
| 5-Hydroxyindole-3-acetic acid | Amino acid derivative | -0.19 (-0.51–0.13) | 0.24 | 0.36 | 0.21 (-0.33–0.75) | 0.45 | 0.79 |
| 5-hydroxytryptophan | Amino acid derivative | 0.19 (-0.11–0.48) | 0.21 | 0.32 |  |  |  |
| Acetylornithine | Amino acid derivative | -0.39 (-0.93–0.14) | 0.15 | 0.24 | -0.2 (-0.77–0.36) | 0.48 | 0.79 |
| Asymmetric dimethylarginine | Amino acid derivative | 0.01 (-0.33–0.35) | 0.97 | 0.98 |  |  |  |
| Betaine | Amino acid derivative | -0.21 (-0.43–0.02) | 0.069 | 0.15 | -0.01 (-0.2–0.17) | 0.89 | 0.93 |
| Carnitine | Amino acid derivative | 0 (-0.16–0.16) | 0.99 | 0.99 | -0.19 (-0.48–0.11) | 0.22 | 0.55 |
| Creatine | Amino acid derivative | -0.25 (-0.45–-0.05) | 0.015 | 0.048 | -0.04 (-0.22–0.15) | 0.68 | 0.84 |
| Creatinine | Amino acid derivative | -0.02 (-0.17–0.12) | 0.75 | 0.84 |  |  |  |
| Cystine | Amino acid derivative | 0.21 (-0.02–0.45) | 0.078 | 0.15 | 0.39 (-0.28–1.06) | 0.25 | 0.55 |
| Guanidinoacetic acid | Amino acid derivative | -0.26 (-0.52–0) | 0.048 | 0.11 |  |  |  |
| Guanidinosuccinic acid | Amino acid derivative | -0.1 (-0.59–0.39) | 0.68 | 0.78 | -0.47 (-1.27–0.33) | 0.24 | 0.55 |
| Hippuric acid | Amino acid derivative | -0.31 (-1–0.38) | 0.38 | 0.52 |  |  |  |
| Histamine | Amino acid derivative | -0.16 (-0.58–0.27) | 0.46 | 0.59 | 1.59 (0.78–2.39) | 0.00019 | 0.0018 |
| Indole | Amino acid derivative |  |  |  | 0.43 (0.24–0.62) | 2.3e-05 | 0.00041 |
| Indole-3-acetic acid | Amino acid derivative | -0.82 (-1.18–-0.46) | 2.5e-05 | 0.00027 | -0.32 (-0.71–0.07) | 0.11 | 0.34 |
| Indole-3-methyl acetic acid | Amino acid derivative | -0.3 (-0.88–0.28) | 0.3 | 0.42 |  |  |  |
| indole-3-propionic acid | Amino acid derivative | 0.15 (-0.41–0.71) | 0.59 | 0.71 | 0.22 (-0.5–0.93) | 0.55 | 0.8 |
| Kynurenic acid | Amino acid derivative | 0.26 (-0.24–0.75) | 0.3 | 0.42 |  |  |  |
| Kynurenine | Amino acid derivative | -0.18 (-0.5–0.14) | 0.26 | 0.38 | 0.49 (-0.15–1.12) | 0.13 | 0.39 |
| Methionine sulfoxide | Amino acid derivative | -0.84 (-1.16–-0.52) | 2.2e-06 | 4.6e-05 | -0.24 (-0.86–0.38) | 0.44 | 0.79 |
| Methylguanidine | Amino acid derivative |  |  |  | 0.19 (-0.5–0.87) | 0.59 | 0.8 |
| Methylhistidin | Amino acid derivative | 0.04 (-0.49–0.56) | 0.89 | 0.94 |  |  |  |
| N-Acetylalanine | Amino acid derivative | 0.33 (0.03–0.63) | 0.031 | 0.077 |  |  |  |
| N-Acetylglycine | Amino acid derivative | 0.76 (0.09–1.43) | 0.028 | 0.073 |  |  |  |
| N-Acetylphenylalanine | Amino acid derivative | -0.48 (-0.76–-0.19) | 0.0014 | 0.0068 |  |  |  |
| N-acetyltryptophan | Amino acid derivative | -0.36 (-0.75–0.04) | 0.074 | 0.15 |  |  |  |
| N,N,N-Trimethyllysine | Amino acid derivative | -0.2 (-0.43–0.04) | 0.1 | 0.19 | 0.19 (0–0.38) | 0.049 | 0.24 |
| Pyroglutamic acid | Amino acid derivative | -0.02 (-0.17–0.12) | 0.73 | 0.83 | 0.03 (-0.12–0.18) | 0.67 | 0.84 |
| Quinolinic acid | Amino acid derivative | 0.37 (0.04–0.71) | 0.031 | 0.077 |  |  |  |
| Serotonin | Amino acid derivative | 0.06 (-0.69–0.81) | 0.87 | 0.94 |  |  |  |
| Glycochenodeoxycholic acid | Bile acid | -1.7 (-2.39–-1.01) | 6.4e-06 | 8.5e-05 |  |  |  |
| Glycocholic acid | Bile acid | -1.26 (-1.94–-0.58) | 0.00046 | 0.0026 | 0.35 (-0.25–0.95) | 0.24 | 0.55 |
| Glycodeoxycholic acid | Bile acid | -1.96 (-2.92–-1.01) | 0.00012 | 0.00097 | 0.16 (-0.9–1.22) | 0.76 | 0.87 |
| Glycohyocholic acid | Bile acid | -0.7 (-1.38–-0.02) | 0.044 | 0.1 |  |  |  |
| Glycohyodeoxycholic acid | Bile acid | -0.73 (-1.6–0.13) | 0.094 | 0.18 |  |  |  |
| Taurocholic acid | Bile acid | -1.62 (-2.6–-0.63) | 0.0017 | 0.0073 |  |  |  |
| Taurodeoxycholic acid | Bile acid | -1.54 (-2.29–-0.78) | 0.00013 | 0.00098 |  |  |  |
| Acetylcarnitine | Lipid | 0.39 (0.06–0.73) | 0.023 | 0.065 |  |  |  |
| Decanoylcarnitine | Lipid | 0.3 (-0.14–0.73) | 0.18 | 0.28 |  |  |  |
| Eicosapentaenoic acid | Lipid | 0.16 (-0.23–0.55) | 0.42 | 0.56 | 0.94 (0.5–1.38) | 6.4e-05 | 0.00078 |
| Ethanolamine | Lipid |  |  |  | 0.03 (-0.21–0.28) | 0.78 | 0.88 |
| Glutarylcarnitine | Lipid | -0.12 (-0.51–0.26) | 0.52 | 0.66 | 3.06 (1.5–4.62) | 2e-04 | 0.0018 |
| Hexanoylcarnitine | Lipid | 0.27 (-0.08–0.63) | 0.13 | 0.21 |  |  |  |
| Isobutyrylcarnitine | Lipid | -0.4 (-0.71–-0.09) | 0.013 | 0.044 | 0.59 (0.12–1.06) | 0.015 | 0.09 |
| Isovalerylcarnitine | Lipid | -0.71 (-1–-0.43) | 4.3e-06 | 6.5e-05 | 0.26 (-0.02–0.55) | 0.071 | 0.27 |
| Lauroylcarnitine | Lipid | 0.1 (-0.28–0.49) | 0.59 | 0.71 |  |  |  |
| Linoleic acid | Lipid | -0.11 (-0.51–0.29) | 0.58 | 0.71 | 0.26 (-0.04–0.55) | 0.086 | 0.29 |
| Octanoylcarnitine | Lipid | 0.35 (-0.08–0.78) | 0.11 | 0.2 |  |  |  |
| Palmitoylcarnitine | Lipid | 0.21 (0.03–0.39) | 0.025 | 0.068 |  |  |  |
| PE 15:0-18:1 | Lipid | -0.35 (-0.52–-0.17) | 0.00018 | 0.0013 |  |  |  |
| Propionylcarnitine | Lipid | -0.63 (-0.92–-0.33) | 6.8e-05 | 6e-04 |  |  |  |
| Sphingomyelin | Lipid | -0.33 (-1.22–0.56) | 0.46 | 0.59 |  |  |  |
| 4-Pyridoxic acid | Nucleotide or its derivatives | -0.19 (-0.53–0.16) | 0.28 | 0.4 |  |  |  |
| Adenine | Nucleotide or its derivatives | -0.1 (-0.51–0.32) | 0.65 | 0.76 | -1.3 (-1.76–-0.84) | 3.3e-07 | 1.2e-05 |
| Cytidine | Nucleotide or its derivatives | 0.08 (-0.49–0.64) | 0.78 | 0.86 | 0 (-0.13–0.13) | 1 | 1 |
| Cytosine | Nucleotide or its derivatives | -1.1 (-1.47–-0.73) | 1.3e-07 | 3.3e-06 |  |  |  |
| Dihydrouracil | Nucleotide or its derivatives | -0.28 (-0.46–-0.1) | 0.0028 | 0.011 | -0.11 (-0.3–0.07) | 0.23 | 0.55 |
| Guanosine | Nucleotide or its derivatives |  |  |  | 0.05 (-0.45–0.55) | 0.85 | 0.93 |
| Hypoxanthine | Nucleotide or its derivatives | 0.03 (-0.18–0.24) | 0.77 | 0.85 | -0.05 (-0.27–0.17) | 0.66 | 0.84 |
| Inosine | Nucleotide or its derivatives |  |  |  | 0.09 (-0.11–0.3) | 0.36 | 0.74 |
| Methylthioadenosine | Nucleotide or its derivatives | -0.18 (-0.45–0.08) | 0.18 | 0.28 | 0.73 (0.44–1.02) | 4.1e-06 | 1e-04 |
| Orotic acid | Nucleotide or its derivatives |  |  |  | -0.1 (-0.23–0.03) | 0.13 | 0.39 |
| Thymine | Nucleotide or its derivatives | -0.34 (-0.62–-0.06) | 0.019 | 0.056 |  |  |  |
| Uracil | Nucleotide or its derivatives | 0.55 (0.31–0.8) | 2.4e-05 | 0.00027 |  |  |  |
| Uric acid | Nucleotide or its derivatives |  |  |  | 0.23 (-0.11–0.56) | 0.19 | 0.52 |
| Xanthine | Nucleotide or its derivatives | 0.21 (0–0.43) | 0.053 | 0.11 |  |  |  |
| 2-Deoxy-D-Glucose | Other | -0.18 (-0.9–0.53) | 0.61 | 0.72 |  |  |  |
| 3-Amino-4-Hydroxybenzoic acid | Other | 0.36 (-0.07–0.79) | 0.097 | 0.18 |  |  |  |
| Biliverdin | Other | 0.37 (0.07–0.68) | 0.018 | 0.056 | -0.22 (-0.88–0.44) | 0.51 | 0.8 |
| Cadaverine | Other | 0 (-0.03–0.03) | 0.96 | 0.98 |  |  |  |
| Carnosine | Other | -0.5 (-1.44–0.44) | 0.29 | 0.42 |  |  |  |
| Choline | Other |  |  |  | -0.09 (-0.32–0.15) | 0.46 | 0.79 |
| Citric acid | Other |  |  |  | -0.41 (-1.13–0.3) | 0.25 | 0.55 |
| Deoxycarnitine | Other | -0.32 (-0.54–-0.1) | 0.0058 | 0.02 | -0.1 (-0.46–0.26) | 0.59 | 0.8 |
| Hypotaurine | Other | 0.03 (-0.6–0.65) | 0.93 | 0.98 |  |  |  |
| Malic acid | Other | -0.45 (-0.63–-0.28) | 2.8e-06 | 5e-05 | -0.09 (-0.37–0.2) | 0.55 | 0.8 |
| N-Acetylputrescine | Other | -0.12 (-0.3–0.06) | 0.19 | 0.3 | 0.21 (-0.11–0.53) | 0.2 | 0.54 |
| Paraxanthine | Other | -0.63 (-1.16–-0.09) | 0.022 | 0.065 | 0.77 (-0.09–1.63) | 0.079 | 0.28 |
| Phenylacetaldehyde | Other | -0.26 (-0.52–0) | 0.047 | 0.11 | 0.91 (0.51–1.32) | 2.8e-05 | 0.00042 |
| Phosphonoacetate | Other | 0.25 (0.03–0.47) | 0.03 | 0.077 |  |  |  |
| Spermidine | Other | 0.3 (-0.09–0.69) | 0.13 | 0.21 | -0.14 (-0.52–0.24) | 0.47 | 0.79 |
| Trigonelline | Other | -1.22 (-2.06–-0.39) | 0.0048 | 0.017 | 0.56 (-0.44–1.57) | 0.27 | 0.56 |
| Trimethylamine-N-Oxide | Other | -0.37 (-0.84–0.1) | 0.12 | 0.21 | -0.56 (-1.15–0.03) | 0.062 | 0.27 |
| Hexose (sum of hexoses) | Sugar or its derivatives |  |  |  | 0.19 (-0.58–0.96) | 0.63 | 0.83 |
| N-Acetylglucosamine | Sugar or its derivatives |  |  |  | 0.53 (0.2–0.86) | 0.0018 | 0.015 |
| N-Acetylneuraminate | Sugar or its derivatives |  |  |  | 0.32 (0–0.64) | 0.052 | 0.24 |
| Biotin | Vitamin | -0.39 (-0.61–-0.18) | 0.00054 | 0.0029 | 0.59 (0.13–1.05) | 0.013 | 0.085 |
| Niacinamide | Vitamin | 0.01 (-0.32–0.33) | 0.97 | 0.98 | -0.78 (-1.31–-0.25) | 0.0042 | 0.031 |
| Pantothenic acid | Vitamin | 0.07 (-0.2–0.34) | 0.58 | 0.71 | 0.37 (0.07–0.68) | 0.017 | 0.091 |
| Retinol | Vitamin | -0.21 (-0.45–0.03) | 0.088 | 0.17 |  |  |  |
| Riboflavin | Vitamin | 0.03 (-0.4–0.47) | 0.88 | 0.94 |  |  |  |
| Thiamine | Vitamin | -0.6 (-1.26–0.05) | 0.072 | 0.15 | -0.06 (-1.5–1.38) | 0.93 | 0.96 |

Supplementary Table S3. Results from pathway analysis for serum and CSF metabolites significantly altered between OA and HC or NINS, respectively.

| **Matrix** | **Pathway** | **Total** | **Expected** | **Hits** | **Raw p** | **-log10(p)** | **Holm adjust** | **FDR** | **Impact** |
| --- | --- | --- | --- | --- | --- | --- | --- | --- | --- |
| **Serum** | Valine, leucine and isoleucine biosynthesis | 8 | 0.11055 | 4 | 1.8517e-06 | 5.7324 | 0.00014813 | 0.00014813 | 0 |
|  | Arginine and proline metabolism | 36 | 0.49749 | 4 | 0.0012067 | 2.9184 | 0.095327 | 0.048267 | 0.20581 |
|  | Pantothenate and CoA biosynthesis | 20 | 0.27638 | 3 | 0.0022445 | 2.6489 | 0.17507 | 0.051903 | 0.02721 |
|  | beta-Alanine metabolism | 21 | 0.2902 | 3 | 0.0025951 | 2.5858 | 0.19983 | 0.051903 | 0.05597 |
|  | Valine, leucine and isoleucine degradation | 40 | 0.55276 | 3 | 0.016255 | 1.789 | 1 | 0.26009 | 0 |
|  | Primary bile acid biosynthesis | 46 | 0.63568 | 3 | 0.023669 | 1.6258 | 1 | 0.31558 | 0.02587 |
|  | Phenylalanine, tyrosine and tryptophan biosynthesis | 4 | 0.055276 | 1 | 0.054191 | 1.2661 | 1 | 0.55484 | 0.5 |
|  | Alanine, aspartate and glutamate metabolism | 28 | 0.38693 | 2 | 0.055484 | 1.2558 | 1 | 0.55484 | 0 |
|  | Glycine, serine and threonine metabolism | 33 | 0.45603 | 2 | 0.074362 | 1.1286 | 1 | 0.661 | 0 |
|  | Pyrimidine metabolism | 39 | 0.53894 | 2 | 0.099328 | 1.0029 | 1 | 0.6654 | 0.07756 |
|  | Phenylalanine metabolism | 8 | 0.11055 | 1 | 0.10557 | 0.97645 | 1 | 0.6654 | 0 |
|  | Taurine and hypotaurine metabolism | 8 | 0.11055 | 1 | 0.10557 | 0.97645 | 1 | 0.6654 | 0 |
|  | Tryptophan metabolism | 41 | 0.56658 | 2 | 0.10813 | 0.96607 | 1 | 0.6654 | 0.14305 |
|  | Biotin metabolism | 10 | 0.13819 | 1 | 0.13025 | 0.88521 | 1 | 0.7443 | 0.2 |
|  | Arginine biosynthesis | 14 | 0.19347 | 1 | 0.17768 | 0.75037 | 1 | 0.94761 | 0.06383 |
|  | Histidine metabolism | 16 | 0.22111 | 1 | 0.20045 | 0.69799 | 1 | 1 | 0.22131 |
|  | Ubiquinone and other terpenoid-quinone biosynthesis | 19 | 0.26256 | 1 | 0.23349 | 0.63173 | 1 | 1 | 0 |
|  | Selenocompound metabolism | 20 | 0.27638 | 1 | 0.24421 | 0.61223 | 1 | 1 | 0 |
|  | Citrate cycle (TCA cycle) | 20 | 0.27638 | 1 | 0.24421 | 0.61223 | 1 | 1 | 0.04412 |
|  | Pyruvate metabolism | 23 | 0.31784 | 1 | 0.27552 | 0.55984 | 1 | 1 | 0.0283 |
|  | Glutathione metabolism | 28 | 0.38693 | 1 | 0.32497 | 0.48816 | 1 | 1 | 0 |
|  | Lysine degradation | 30 | 0.41457 | 1 | 0.34383 | 0.46365 | 1 | 1 | 0.00204 |
|  | Glyoxylate and dicarboxylate metabolism | 32 | 0.44221 | 1 | 0.36219 | 0.44106 | 1 | 1 | 0 |
|  | Tyrosine metabolism | 42 | 0.5804 | 1 | 0.44687 | 0.34982 | 1 | 1 | 0.13972 |
| **CSF** | Phenylalanine metabolism | 8 | 0.035176 | 1 | 0.034714 | 1.4595 | 1 | 1 | 0.14286 |
|  | Nicotinate and nicotinamide metabolism | 15 | 0.065955 | 1 | 0.064237 | 1.1922 | 1 | 1 | 0.1943 |
|  | Histidine metabolism | 16 | 0.070352 | 1 | 0.068391 | 1.165 | 1 | 1 | 0.18852 |
|  | Cysteine and methionine metabolism | 33 | 0.1451 | 1 | 0.13662 | 0.86447 | 1 | 1 | 0.02089 |
|  | Biosynthesis of unsaturated fatty acids | 36 | 0.15829 | 1 | 0.14821 | 0.82912 | 1 | 1 | 0 |
|  | Amino sugar and nucleotide sugar metabolism | 42 | 0.18467 | 1 | 0.17098 | 0.76705 | 1 | 1 | 0.07184 |
|  | Purine metabolism | 70 | 0.30779 | 1 | 0.27048 | 0.56786 | 1 | 1 | 0.01908 |
